# Genomic Evidence for Mobile-Element-Associated Resistance: Predicted MOBH-Family Relaxase Sharing and Adjacent ICE-Cassette Architectures in the *Pseudomonas guariconensis* Clade

**DOI:** 10.3390/microorganisms14071428

**Published:** 2026-06-30

**Authors:** Fuad Alanazi, Abdulhadi M. Abdulwahed, Abdulrahman Alrezaihi, Mohammed Ali M. Marie, Alanoud T. Aljasham, Raed Farzan

**Affiliations:** Department of Clinical Laboratory Sciences, College of Applied Medical Sciences, King Saud University, Riyadh 12372, Saudi Arabia

**Keywords:** putida group, comparative genomics, carbapenemase, metallo-β-lactamase, integrative conjugative element, tick microbiome, *Hyalomma dromedarii*, *Pseudomonas shiyinii*, One Health, wastewater

## Abstract

We analysed nine *Pseudomonas guariconensis*-clade genomes from environmental and clinical sources across four continents to test whether carriage of acquired resistance is associated with the acquisition of mobile elements. FA-1, our tick-derived anchor from *Hyalomma dromedarii* on Saudi camels, sits at 87.52–87.94% ANI to the other genomes on the longest external branch of the core-genome ML phylogeny. In the current NCBI type-strain ANI taxonomy check, FA-1 is conspecific with the recently described *Pseudomonas shiyinii* type strain ST4 (98.05% ANI). The recently reported Vietnamese hospital-wastewater isolate KNHN1 groups within the sensu stricto clinical clade alongside USA-Nashville and India at 99.7%/99% (SH-aLRT/UFBoot) support. Across the nine genomes, three distinct carbapenemase architectures emerged: Dao (chromosomal KPC-2 + NDM-1 + AFM-5), Ethiopia (chromosomal NDM-1 × 2 + plasmid VIM-4), and Korea (plasmid VIM-2). Dao and Ethiopia chromosomes share a MOBH-family relaxase signal, extending the MOBH marker to the clinical compartment. MBL-positive genomes carried more mobile elements (Dao 55; Ethiopia 22; Korea 16; 16–55 hits) than MBL-negative comparators (0–8 hits), an unadjusted descriptive observation (*n* = 3 vs. 6). Ethiopia presented an integron-rich chromosomal and plasmid architecture (six structures), while Dao carried IS-bounded transposon islands on the chromosome. Three of four Dao chromosomal ICE predictions and one Ethiopia ICE prediction lay 27–50 kb from IS-bounded carbapenemase loci; a fourth Dao ICE was standalone and the major Ethiopia NDM-1 + OXA-10 cassette lacked an adjacent complete ICE prediction, indicating descriptive ICE-cassette proximity rather than functionally demonstrated mobility. Together, these findings are consistent with mobile-element acquisition as a plausible route for the emergence of clinical resistance within this clade, with environmental relatives retaining empty insertion sites at the AMR-cassette locus.

## 1. Introduction

*Pseudomonas guariconensis* was originally described from Venezuelan rhizosphere soil as a plant-associated environmental species [1]. Over the past three years, however, *P. guariconensis* has been increasingly reported in clinically significant human-infection contexts: a Tunisian hospital reported an OXA-204 carbapenemase carried on a 119 kb chromosomally integrated plasmid fragment from a 2023 case (published 2025) [2]; a Japanese laboratory reported the first post-traumatic bacteraemia case following agricultural-machinery injury, with an elevated meropenem MIC of 8 mg/L, in the same year [3]; a Danish necrotising soft-tissue infection followed shortly after [4]; and additional clinical isolates have now been deposited from urine, pleural fluid, blood, and wound sites across Korea, India, Mexico, Ethiopia, and the USA. The contrast between the species’ described environmental origin and these emerging clinical reports raises a mechanistic question that the existing clinical literature cannot address in isolation: whether the genotypically predicted acquired-resistance phenotype observed in some clinical isolates is driven by features intrinsic to the *P. guariconensis* lineage, or by horizontal acquisition of mobile resistance determinants on top of an otherwise susceptible environmental backbone.

Answering this question requires a paired comparison between clinical and environmental *P. guariconensis*-clade genomes from a metadata-explicit comparative genome panel spanning environmental and clinical sources. The published environmental literature for the species is sparse: a single Nigerian soil genome reported in 2025 (ABJ_B2_1) [5], which in our uniform screen carried only intrinsic Mex-family efflux pumps and no acquired resistance genes, the original Venezuelan type strain PCAVU11T (=LMG 27394^T^) [1], a Vietnam isolate from urban wastewater near a major hospital carrying experimentally self-transmissible ICE-borne carbapenemases [6], and surveys of camel-tick microbiomes dominated by *Francisella* and *Corynebacterium* with no *P. guariconensis*-clade detection [7]. Khogali [8] similarly reported *Francisella* dominance and *Pseudomonas* detected only at the genus level in Kenyan *H. dromedarii*; together, these tick surveys yielded no isolate-level whole-genome data for the *P. guariconensis* clade, while broader *Pseudomonas* phylogenomic studies illustrate the importance of genome-resolved species delimitation [9]. To our knowledge, no genome of an arthropod-vector-derived isolate from this lineage has been described, leaving a gap in the comparative reference set that prevents formal testing of the intrinsic-versus-acquired resistance hypothesis.

Based on the broader literature on environmental-to-clinical antimicrobial-resistance exchange in *Pseudomonas*, including documented mobile-element exchanges between rhizosphere, soil, water, and hospital reservoirs, and on the recent demonstration of self-transmissible ICE-borne carbapenemases in a wastewater isolate of this clade [6], we hypothesised a priori that the resistome architecture and chromosomal mobile-element context of arthropod-vector environmental isolates would differ systematically from clinical members of the *P. guariconensis* clade in both gene content and the chromosomal scaffold on which acquired resistance determinants reside.

Here we sequenced and assembled FA-1, a *Pseudomonas* isolate recovered from the internal tissues of *Hyalomma dromedarii* ticks collected from dromedary camels at the Hail Camels’ Market, Saudi Arabia, in a cross-sectional bacterial survey of camel-tick AMR carriers [10]. FA-1 fell at 87.5–87.9% ANI to all known *P. guariconensis* genomes, placing it outside *Pseudomonas guariconensis* sensu stricto; in the current NCBI type-strain ANI taxonomy check, FA-1 is conspecific with the recently described *Pseudomonas shiyinii* [11] (98.05% ANI), and is therefore reframed here as a tick-derived *isolate assigned to P. shiyinii based on ANI*. We then assembled a focused nine-genome dataset (including the recently published Dao et al. Vietnam wastewater isolate KNHN1 [6] as a second environmental MBL-positive reference), comprising FA-1, the *P. guariconensis* type strain, the Nigerian soil reference, and five clinical genomes spanning three continents: HY1196 from Korean urine in 2023, MGL_R1_GC from Indian pleural fluid in 2022, GN-165 from Mexican blood culture in 2022, PM99 from Ethiopian urine in 2023, and SAR321 from a USA wound in 2025; we then tested the central hypothesis: whether acquired antimicrobial resistance distributions and the chromosomal contexts of metallo-β-lactamase genes differ systematically between clinical and environmental members of the clade.

## 2. Materials and Methods

### 2.1. Tick Collection, Culture, and DNA Preparation

This was a retrospective comparative genomic analysis anchored by one newly sequenced isolate (FA-1) from an a prior cross-sectional tick survey [10] and eight publicly available comparator genomes. *Hyalomma dromedarii* ticks were collected from 13 female dromedary camels at the Hail Camels’ Market, Hail Province, Saudi Arabia, between October and November of the upstream collection campaign, with verbal consent from camel owners and following local institutional guidelines for non-invasive external-parasite removal from livestock; no experimental manipulation or invasive procedures were performed on the camels, and formal IRB approval was not required for non-invasive arthropod collection from client-owned animals [10]. Tick processing followed the upstream protocol [10]: surface sterilisation in 70% ethanol, manual homogenisation, enrichment in nutrient broth, and plating on blood agar and MacConkey agar. A *Pseudomonas* isolate (designated FA-1 in this study) was recovered from this tick collection and advanced to whole-genome sequencing (WGS). The upstream survey used VITEK 2 GN biochemical screening and antimicrobial susceptibility testing for isolates from this collection [10]; however, because isolate-specific phenotypic testing was not performed for FA-1 in this work, and the exact colony-level linkage to the VITEK-tested isolates cannot be verified, those data are treated here as collection-level context rather than per-isolate phenotypic validation. Species-level assignment in the present study is therefore based on genome-wide ANI rather than biochemical identification, particularly because *P. guariconensis* is not represented in the VITEK 2 GN database. Genomic DNA was subsequently extracted from the recovered isolate using a CTAB protocol. Briefly, bacterial biomass was lysed, treated with CTAB/NaCl and proteinase K, extracted with chloroform–isoamyl alcohol, precipitated with isopropanol, washed with ethanol, and resuspended in TE buffer. The extracted DNA was submitted to Macrogen, Inc. (Seoul, Republic of Korea) for Illumina (San Diego, CA, USA) shotgun sequencing.

### 2.2. Sequencing, Assembly, and Quality Assessment

A preliminary shotgun dataset (SRA SRR38121107), generated in the upstream workflow from the same cultured-isolate DNA as the full WGS run (SRR38121108), was used for lineage-level taxonomic profiling with MetaPhlAn4 [12] (mpa_vJun23_CHOCOPhlAnSGB_202403 database) and Kraken2 v2.1 [13] (PlusPFP database) and served as an orthogonal pre-WGS culture-purity check (Supplementary File S1). Because FA-1, now interpreted as a *Pseudomonas shiyinii*-related lineage, was absent from the default Kraken2 reference database, reads may be assigned by the lowest common ancestor (LCA) algorithm to the nearest available *P. guariconensis*/*P. putida*-group labels; profiling outputs were therefore interpreted as database-dependent lineage-level bins rather than as evidence for a distinct co-coloniser. The isolated colony was advanced to the full isolate WGS run (SRA SRR38121108); reads were assembled with SPAdes v3.15.5 [14] using default parameters with contigs ≥500 bp retained, and assembly quality and completeness were assessed with QUAST v5.2.0 [15], BUSCO v5.7.1 [16] (bacteria_odb10), and CheckM2 v1.1.0 [17] (Neural Network specific model, uniref100.KO DIAMOND database). The assembly was deposited at DDBJ/ENA/GenBank under accession JBWXTH000000000; the version described in this paper is version JBWXTH010000000 (BioProject PRJNA1449514; BioSample SAMN57124393).

### 2.3. Comparator Genome Selection

To investigate whether the *P. guariconensis* clinical lineage carries metallo-β-lactamase (MBL) resistance genes within mobile genetic element (MGE) architectures that are distinguishable from those of environmental relatives, we assembled a panel of nine *P. guariconensis*-clade genomes spanning environmental and clinical isolation sources across four continents (Table 1). The panel comprised: one own-assembled environmental anchor (FA-1, this study); one taxonomic anchor (type strain LMG 27394^T^, GCF_900102675.1); one environmental sub-clade representative (Nigerian soil ABJ_B2_1, GCF_044903045.1); one environmental MBL-positive isolate (Dao et al. Vietnam wastewater KNHN1; DDBJ chromosome AP035765.1 + plasmid pKNHN1 AP035766.1) [6]; and five clinical isolates from human infections, Korea HY1196 urine 2023 (GCF_034724015.1), India MGL_R1_GC pleural fluid 2022 (GCF_036528595.1), Mexico GN-165 blood culture 2022 (GCF_036879405.1), Ethiopia PM99 urine 2023 (GCF_051550215.1) and USA-Nashville SAR321 wound 2025 (GCF_052077635.1). The inclusion of the Dao et al. Vietnam wastewater isolate converts the panel from a binary environmental-versus-clinical contrast to a broader comparative framework and provides a second environmental MBL-positive reference against which the Ethiopia chromosomal carbapenemase architecture can be compared. All comparators passed identical contiguity and size quality checks (approximately 5.0–6.3 Mb genome size [reflecting the larger KNHN1 chromosome + plasmid total of ~6.29 Mb]; assemblies in the *Pseudomonas* range; see Supplementary Table S1 for extended per-genome metadata including assembly level, genome size, GC content, and isolation source), and metadata (country, year, host, isolation source, stratum) were curated from the corresponding BioSample records and from the species descriptions where BioSample fields were absent.

### 2.4. Average Nucleotide Identity, Taxonomy, and Phylogeny

All-vs-all average nucleotide identity was computed with FastANI v1.33 [18] across the nine-genome set using default parameters (fragment length 3000 bp, minimum aligned fragments 50). Coding sequences were predicted with Prodigal v2.6.3 [19] in single-genome mode for each assembly. Pan-genome analysis used Panaroo v1.6.1 [20] in strict cleaning mode (--clean-mode strict, --core_threshold 0.95) with MAFFT v7.490 [21] to align core gene clusters. The resulting core-genome alignment (3151 of 3218 core genes after entropy filtering; 29.2 Mb total alignment matrix across the nine taxa, not a per-taxon aligned length) was used as input for a maximum-likelihood phylogeny computed with IQ-TREE 2 v2.3.6 [22] using ModelFinder [23] (option -m MFP), 1000 ultrafast bootstrap replicates [24] and 1000 SH-aLRT replicates [25] (option -B 1000 --alrt 1000). Trees were midpoint-rooted (no external outgroup was included) and rendered with Bio.Phylo; given the long external branch of FA-1, this midpoint rooting is used for visual orientation rather than to make definitive claims about the ancestral root of the clade, and rooting-dependent inferences are interpreted conservatively, with FA-1 treated as a divergent *Pseudomonas shiyinii*-related lineage within the broader *P. guariconensis*-associated comparative framework. Digital DNA–DNA hybridisation (dDDH) was additionally estimated for the FA-1 assembly against type-strain genomes using a TYGS analysis under the GGDC 4.0 algorithm with recommended settings [26,27]; species membership was assessed against the standard 70% dDDH species-delimitation threshold. The resulting genome-based GBDP tree, placing FA-1 among its closest *Pseudomonas* type strains, is provided as Figure S1. Because this analysis was deliberately restricted to the *P. guariconensis* clade, no external outgroup was included. The maximum-likelihood topology was therefore interpreted as an unrooted tree and midpoint-rooted only for visual orientation. The recovered genome groupings and their SH-aLRT/UFBoot support values are properties of the unrooted topology and are not changed by the choice of root; therefore, the inferred relationships among the nine genomes do not depend on midpoint rooting. A distant outgroup was avoided because it could reduce the shared core-genome alignment and introduce long-branch artefacts in this within-clade comparison. Average amino-acid identity (AAI) [28] was additionally estimated across all genome pairs as the mean amino-acid identity over single-copy core orthologues defined by the Panaroo pan-genome, with pairwise protein identities computed by global Needleman–Wunsch alignment under BLOSUM62 in Biopython [29] (Table S6).

### 2.5. Mobile Genetic Element (MGE) Annotation and Typing

Mobile genetic elements were typed using three orthogonal tools to disentangle integron-mediated, integrative-and-conjugative-element (ICE)-mediated, and insertion-sequence (IS)-element-mediated resistance architectures. First, MobileElementFinder v1.1.2 (with mgedb v1.1.1; DTU Center for Genomic Epidemiology) [30] was used for first-pass MGE detection across all nine genomes against its curated database of known MGEs, providing total MGE counts per genome for descriptive comparison of MGE burden between MBL-positive and MBL-negative isolates. Primary MGE burden was reported as unnormalised counts, with genome-size-normalised values (MGE per megabase) additionally computed as a sensitivity check (Section 3.4). Cross-genome MGE-burden comparisons are presented as descriptive contrasts rather than effect-size estimates and may be influenced by differences in assembly completeness across the panel. Second, MOB-suite v3.1.9 [31] was applied to the five selected clinical or carbapenemase-positive genomes (Dao Vietnam wastewater, Ethiopia urine, Korea urine, India pleural, USA-Nashville wound) for relaxase mobility typing and replicon identification via the mob_recon module for plasmid reconstruction followed by mob_typer for replicon and relaxase typing. Third, IntegronFinder v2.0 [32] was run on all nine genomes with the --local-max option for integron detection, providing formal counts of complete integrons, In0 elements (integrase-only structures), and CALIN clusters (clusters of attC sites lacking an integrase). For ICE typing of cassette neighbourhoods, candidate cassette regions were submitted to the ICEfinder web (ICEberg v2.0 database) [33], both as ±10–15 kb FASTA inputs and as full-chromosome FASTA inputs; full-chromosome submissions enabled detection of T4SS-encoding ICEs sitting outside the immediate cassette window. ICEfinder Job IDs are listed in Supplementary Table S4. Conjugation-module completeness was additionally assessed with oriTfinder2 (oriTDB) [34], which detects the origin of transfer (oriT), relaxase, type IV coupling protein (T4CP) and type IV secretion system (T4SS) gene cluster; the two MBL-positive chromosomes (Dao AP035765.1 and Ethiopia NZ_CP194068.1) were submitted under default parameters, and the predicted conjugation modules are shown in Figure S2.

### 2.6. Antimicrobial Resistance Gene Annotation

Antimicrobial resistance genes were initially annotated by Prokka v1.15.6 against the UniProt SwissProt reference set during whole-genome annotation. Allele-level resolution was then obtained by cross-validation with ABRicate v1.4.0 against four AMR-gene databases: CARD [35], NCBI AMRFinderPlus [36], ResFinder [37], and ARG-ANNOT [38]. PlasmidFinder [39] was run separately for plasmid-replicon screening and was not included in the AMR-gene consensus. AMR calls were resolved by ≥80% identity/≥80% coverage thresholds across at least two of the four AMR-gene databases; replicon calls from PlasmidFinder were reported separately. Where Prokka’s UniProt-based annotation differed from the ABRicate consensus, for example, a sequence Prokka labelled *blaNDM-1* that matched at 100% identity to *blaAFM-5* in NCBI AMRFinderPlus, indicating a divergent metallo-β-lactamase from a different family rather than a duplicated NDM-1, the ABRicate consensus call at 100% identity was retained. Substring-based first-pass annotation surfaced two ubiquitous housekeeping CDS, *yrfG* (GMP/IMP nucleotidase) and *yigZ* (IMPACT family), which matched the “IMP” substring; these were excluded by family-membership filtering before downstream analysis. Genes were classified as acquired only after database detection, alias normalisation, and exclusion of known intrinsic *Pseudomonas* background loci (the Mex-family efflux systems and chromosomal regulators removed in Section 2.7). FA-1 was treated as a *P. shiyinii*-related environmental comparator; the conserved species background was defined by the *P. guariconensis* type strain (LMG 27394^T^) together with the other MBL-negative *P. guariconensis* genomes, against which candidate acquired loci were compared. Chromosomal location alone was not treated as evidence of intrinsic origin: chromosomal carbapenemase and OXA-family loci were classified as acquired only when they were absent from this conserved background and embedded in mobile-element/integron/ICE-associated contexts.

### 2.7. Acquired-Resistance Distribution and Statistical Testing

Acquired antimicrobial resistance genes were detected with ABRicate v1.4.0 against four complementary databases, CARD [35] (6052 sequences, 29 April 2026), NCBI AMRFinderPlus [36] (8232), ResFinder [37] (3206), and ARG-ANNOT [38] (2224), using minimum 80% identity and 80% coverage thresholds. PlasmidFinder [39] (488 sequences) was used to screen for *Enterobacterales*-type plasmid replicons. Hits were normalised across databases by stripping ARG-ANNOT class prefixes (e.g., (Bla), (AGly)) and ResFinder version suffixes (_1, _2 …) and merged on normalised gene-name keys. Intrinsic *Pseudomonas* Mex-family efflux pumps, OpmH, MuxABC, TriC, and chromosomal regulators (CpxR, SoxR, RsmA, YajC), were excluded from the acquired-AMR matrix because they are conserved across the genus and not informative for clinical emergence. Fisher’s exact tests were applied to per-stratum (clinical vs. environmental) presence/absence counts using SciPy v1.13 [40] (one-tailed alternative = ‘greater’ for the directional hypothesis (clinical > environmental), two-tailed for transparency). For each 2 × 2 contrast, odds ratios and Wald 95% confidence intervals were estimated, with Haldane–Anscombe correction specified for any zero cell. Given the limited sample size (*n* = 9 genomes), these tests are exploratory and statistical power for cross-stratum contrasts is intrinsically limited at this scale, with the mechanistic conclusions resting on the sequence-resolution synteny evidence rather than on the resistance-frequency contrast alone. Raw first-pass CARD database hits for FA-1 (including subthreshold rows) are provided in Table S2 and the negative-database summary (ResFinder and NCBI AMRFinderPlus) in Table S3; the consolidated nine-genome acquired-resistance presence/absence matrix is presented in Table 2, and the per-allele ABRicate consensus (replicon position, allele identity, and resistance class) in Supplementary Table S5. OXA-10 is a narrow-spectrum class D β-lactamase distinct from the carbapenem-hydrolysing class D β-lactamase (CHDL) lineages (e.g., OXA-23-, OXA-24/40-, OXA-48-, OXA-51- and OXA-58-family enzymes); OXA-10 itself does not hydrolyse carbapenems at clinically relevant levels [41]. The 80%/80% identity/coverage threshold was chosen to capture divergent alleles within the focused nine-genome panel; this is more permissive than the CARD curated-allele default (95%/80%) but appropriate for cross-stratum presence/absence inference where divergent variants of the same gene family are biologically relevant. No multiple-comparison correction was applied because Fisher’s exact testing was restricted to two pre-specified summary endpoints (any acquired-resistance gene; carbapenemase carriage); all per-gene patterns in Table 2 are presented as descriptive rather than inferential.

A lower identity threshold necessarily trades some per-database specificity for sensitivity to divergent alleles; therefore, the 80%/80% cutoff was used as a permissive first-pass screen rather than as a standalone definition of acquired resistance. False-positive calls were constrained by requiring concordance across at least two of the four AMR-gene databases (Section 2.6), by manually removing family-name or substring artefacts such as the housekeeping loci *yrfG* and *yigZ* matching the “IMP” substring, and by reporting raw/subthreshold screening hits and final per-allele consensus support in Tables S2 and S5. For the key carbapenemase-positive genomes, downstream interpretation also used local genomic context and synteny-level inspection, not threshold-dependent presence/absence calls alone.

### 2.8. Mobile Genetic Element Context Analysis

The chromosomal contexts of all metallo-β-lactamase and OXA-family β-lactamase hits identified in clinical genomes were extracted as ±10 kb flanking windows from the source assembly using Biopython v1.84 [29]. Synteny against environmental-comparator genomes was assessed with minimap2 v2.24-r1122 [42] (preset asm10, for low-divergence assembly-to-assembly mapping). Query coverage and alignment-junction positions were extracted from PAF outputs. Two-sided synteny across the circular chromosomal origin was visualised at the gene level with pyGenomeViz v1.6.1 [43], comparing annotated gene order in the Ethiopia cassette region (extended across the origin) with the homologous loci in the type strain and Nigerian soil genomes; the Nigerian track was reverse complemented for display.

### 2.9. Data Availability

This Whole Genome Shotgun project has been deposited at DDBJ/ENA/GenBank under the accession JBWXTH000000000. The version described in this paper is version JBWXTH010000000. Raw reads are deposited under SRA accessions SRR38121108 (full WGS) and SRR38121107 (pre-WGS purity check); BioProject PRJNA1449514; BioSample SAMN57124393. Additional intermediate result files (FastANI matrices, IQ-TREE tree outputs, Panaroo gene-presence–absence tables, ABRicate raw outputs, and minimap2 PAFs) are available from the corresponding authors upon reasonable request.

## 3. Results

### 3.1. FA-1 Is Conspecific with Pseudomonas shiyinii, Outside Pseudomonas guariconensis Sensu Stricto

Taxonomic profiling of the preliminary shotgun dataset supported a high-purity *Pseudomonas* isolate of the *P. guariconensis* lineage. MetaPhlAn4 placed the dominant *Pseudomonas* signal in SGB12273, associated with *P. guariconensis*, and Kraken2 classified 67.09% of total reads (26,721,328/39,831,549), with 96.0% of classified reads assigned to *Pseudomonas* and 33.30% of total reads (13,262,076 reads) assigned to the *P. putida* group; consistent with FA-1’s absence from the default reference database, this *P. putida*-group fraction is interpreted as a database-dependent bin that may include mis-binned FA-1 reads rather than a distinct co-coloniser. The full isolate assembly comprised 38 contigs ≥500 bp, total length 5,098,887 bp, N50 = 363,489 bp, GC = 63.75%, with 99.2% complete BUSCOs (123/124; fragmented 0.8%, missing 0.0%) and, by CheckM2, 100.0% completeness with 0.1% contamination, indicating a high-purity isolate assembly.

**Figure 1:**
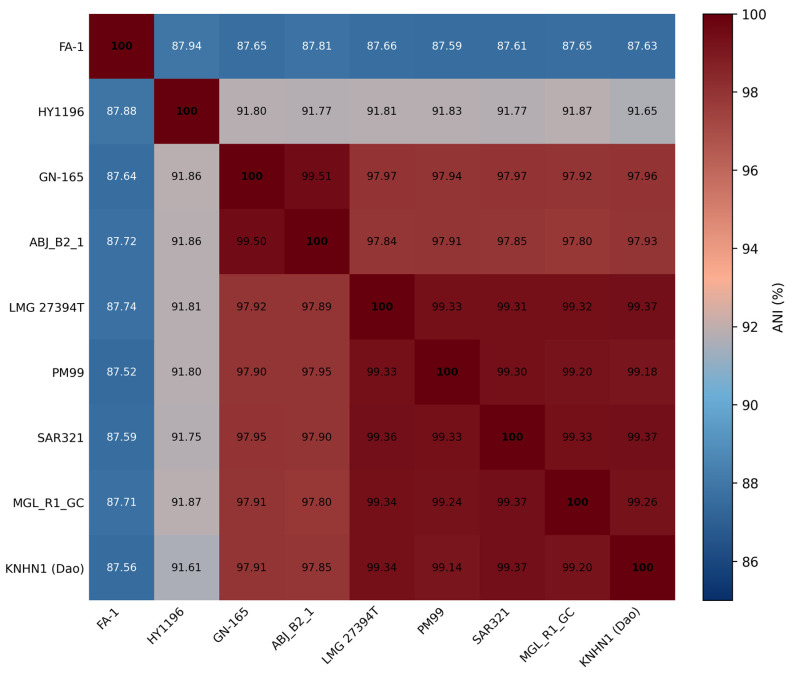
Pairwise FastANI [18] percent-identity heatmap across the nine-genome *Pseudomonas guariconensis*-clade panel. The 9 × 9 matrix shows all-vs-all average nucleotide identity values. The FA-1 tick-vector isolate (this study) sits at 87.52–87.94% identity to every other genome, comfortably below the conventional 95–96% species threshold, consistent with placement outside *P. guariconensis* sensu stricto and with the current assignment of FA-1 as a *Pseudomonas shiyinii*-related isolate. Of the remaining eight genomes, seven cluster within a single species (97.80–99.51% intra-cluster identity), while Korea HY1196 sits at an intermediate 91.61–91.87% position, below the conventional 95% species threshold and consistent with a borderline intra-clade/candidate sister-genomospecies lineage. The Dao et al. [6] Vietnam wastewater isolate (KNHN1) groups within the sensu stricto cluster at 99.26% mean identity. Colour scale: blue-to-red gradient (blue ≈ 86%, white ≈ 92%, red ≈ 100%) indicates increasing identity. FastANI is asymmetric by design; ranges quoted include both query→reference directions. Abbreviations: ANI, average nucleotide identity.

**Figure 2:**
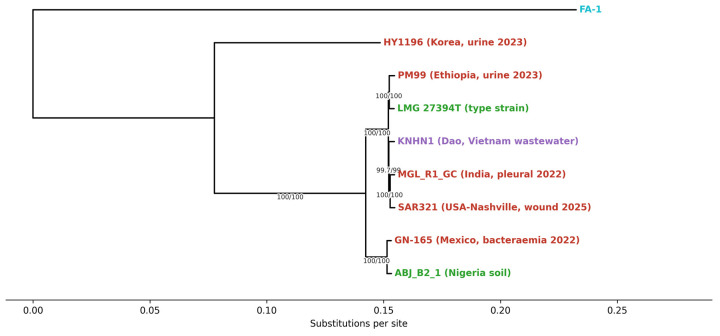
Core-genome maximum-likelihood phylogeny of the nine-genome *Pseudomonas guariconensis*-clade panel. Tree computed by IQ-TREE 2 v2.3.6 [22] on a 3151-of-3218-core-gene alignment (29.2 Mb concatenated matrix) under the GTR + F + I + R7 model with 1000 ultrafast bootstrap replicates and 1000 SH-aLRT replicates. FA-1 (this study) is recovered on the longest external branch (length 0.2324); the tree is midpoint-rooted for visual orientation, and the exact ancestral root remains provisional. Internal bipartitions are uniformly well supported, as detailed below. The Dao et al. Vietnam wastewater isolate (KNHN1) [6] groups within the sensu stricto clinical clade alongside the USA-Nashville wound and India pleural isolates at 99.7% SH-aLRT and 99% ultrafast-bootstrap support. The type-strain (LMG 27394^T^)–Ethiopia (PM99) sister pair carries 100% SH-aLRT and 100% ultrafast-bootstrap support; the (USA-Nashville, India) + Dao trio (carrying SH-aLRT 99.7%/UFBoot 99%) is the only internal bipartition below maximum support. Branch lengths represent substitutions per site. Abbreviations: GTR + F + I + R7, general time-reversible substitution model with empirical base frequencies, invariant sites, and seven free-rate categories (selected by ModelFinder [23]); UFBoot, ultrafast bootstrap; SH-aLRT, Shimodaira–Hasegawa-like approximate likelihood ratio test.

All-vs-all FastANI placed FA-1 between 87.52% and 87.94% ANI to every other genome in the nine-genome set (Figure 1; Table 1), with the closest match (HY1196, Korea urine, 87.94%) comfortably below the conventional 95–96% species threshold. Of the remaining eight genomes, seven clustered tightly within *P. guariconensis* sensu stricto, whereas HY1196 is treated here as a borderline intra-clade/candidate sister-genomospecies rather than as part of the sensu stricto species: this seven-genome sensu stricto cluster, comprising the *P. guariconensis* type strain LMG 27394^T^, the Nigerian soil isolate, the Mexico blood culture isolate, the India pleural isolate, the Ethiopia PM99 urine isolate, the USA-Nashville wound isolate, and the Dao Vietnam wastewater isolate, showed 97.80–99.51% pairwise ANI. The closest pair within this sensu stricto cluster, Mexico ↔ Nigeria, was 99.50% ANI, highlighting genomic continuity between recent clinical isolates and environmental reservoirs within the sensu stricto species. HY1196 sat at 91.61–91.87% ANI to all other clade members except the more divergent FA-1 anchor (~87.9%), intermediate between the species threshold (95%) and the genus-level lower bound (~80%); this supports a provisional borderline intra-clade/candidate sister-genomospecies designation rather than species-level continuity with *P. guariconensis* sensu stricto. This species-level distinction was independently corroborated by digital DNA–DNA hybridisation: a TYGS analysis of the FA-1 assembly returned only 32.9% dDDH (GGDC formula d4; 95% CI 30.4–35.4%) to its closest type strain, *P. guariconensis* LMG 27394^T^, far below the 70% species-delimitation threshold (additional GGDC formulas d0 = 61.6% and d6 = 53.9% likewise below threshold), and assigned FA-1 to a species cluster distinct from *P. guariconensis* sensu stricto. The current NCBI type-strain ANI taxonomy check places FA-1 with the recently described *Pseudomonas shiyinii* type strain ST4 (GCA_056725765.1; 98.05% ANI), supporting reassignment of FA-1 as a *P. shiyinii*-related isolate. Figure 1 and Figure 2 show the nine-genome *P. guariconensis*-clade comparative panel used for the AMR/MGE analyses; ST4 was not part of that panel, so FA-1’s relationship to ST4 was assessed separately through the NCBI type-strain ANI taxonomy check. GTDB-Tk was not run on the nine-genome panel; taxonomic placement therefore rests on all-vs-all FastANI, the core-genome maximum-likelihood phylogeny, dDDH, and the current NCBI type-strain ANI taxonomy check. Average amino-acid identity (AAI) across 3197 Panaroo single-copy core orthologues reproduced the ANI rank order: the seven *P. guariconensis* sensu stricto genomes shared 99.58% mean pairwise AAI (range 99.34–99.83%), whereas HY1196 averaged 97.15% to the sensu stricto cluster and FA-1 averaged 93.57%. Thus, AAI independently supports HY1196 as the most divergent sensu lato genome after FA-1, while its value above the ~95% AAI species heuristic supports retaining the cautious “borderline/provisional” framing rather than treating it as a definitive separate species. FA-1’s lower AAI (93.57%) parallels its lower ANI and supports its divergence from *P. guariconensis* sensu stricto.

Consistent with the FastANI placement, the core-genome maximum-likelihood phylogeny computed on 3218 core genes (3151 after entropy filtering; 29.2 Mb concatenated alignment; best-fit model GTR + F + I + R7) recovered FA-1 on the single longest external branch (branch length 0.2324), outside a tight cluster comprising the *P. guariconensis* type strain, Nigerian soil, and the four clinical isolates other than Korea (Figure 2). Korea HY1196’s isolated long branch (0.0709), sitting outside the tight species-level cluster but inside the broader clade, is consistent with its borderline intra-clade/candidate sister-genomospecies position established earlier in this section, mirroring its FastANI placement. Within the tight cluster the Mexico–Nigeria sister relationship (branches 0.0017/0.0019) was resolved with maximum support; five of the six internal bipartitions (the only nontrivial splits in an unrooted nine-leaf binary tree) carried maximum (100/100) SH-aLRT and ultrafast-bootstrap support, including the type-strain–Ethiopia (PM99) sister relationship (branches 0.0020/0.0023); the sixth, the (USA-Nashville, India) + Dao clade described below, carried 99.7%/99% (SH-aLRT/UFBoot) support, indicating an unambiguously resolved phylogenetic backbone. When the Dao et al. Vietnam wastewater isolate KNHN1 (AP035765 + AP035766) was added to the panel, it grouped within the sensu stricto clade alongside the USA-Nashville wound and India pleural clinical isolates at 99.7% SH-aLRT and 99% UFBoot support; FastANI confirmed Dao’s mean ANI to the sensu stricto reference cluster (type strain, USA-Nashville, India, Ethiopia) at 99.26%, placing this environmental wastewater isolate among the clinical sensu stricto strains rather than with the more divergent environmental sub-clades (Dao vs. Mexico–Nigeria sub-clade, ~97.9% ANI; Dao vs. Korea, ~91.7% ANI; Dao vs. FA-1, ~87.6% ANI).

### 3.2. Pan-Genome Architecture of the Focused Clade

Panaroo identified 8402 unique gene clusters across the nine-genome dataset, of which 3218 were core (present in 99–100% of strains), 2159 were shell (15–95%), and 3025 were cloud genes (<15%). The absence of a soft-core fraction (95–99% strains) is an inevitable binning artefact at *n* = 9 because the 95–99% range corresponds to a non-integer 8.55–8.91 strains; the high cloud-gene burden (3025/8402 = 36%) is consistent with substantial accessory diversity at the focused-clade scale (acknowledging that *n* = 9 and FA-1’s *P. shiyinii*-related status limit pan-genome openness inference), dominated by accessory content rather than a shared accessory genome. The largest per-genome accessory contribution came from the Ethiopia PM99 urine isolate, which carried a disproportionate share of cloud genes consistent with the additional resistance-cassette machinery described below (Section 3.3 and Section 3.4).

### 3.3. Acquired Antimicrobial Resistance Is Restricted to a Subset of Clinical Isolates

**Figure 3:**
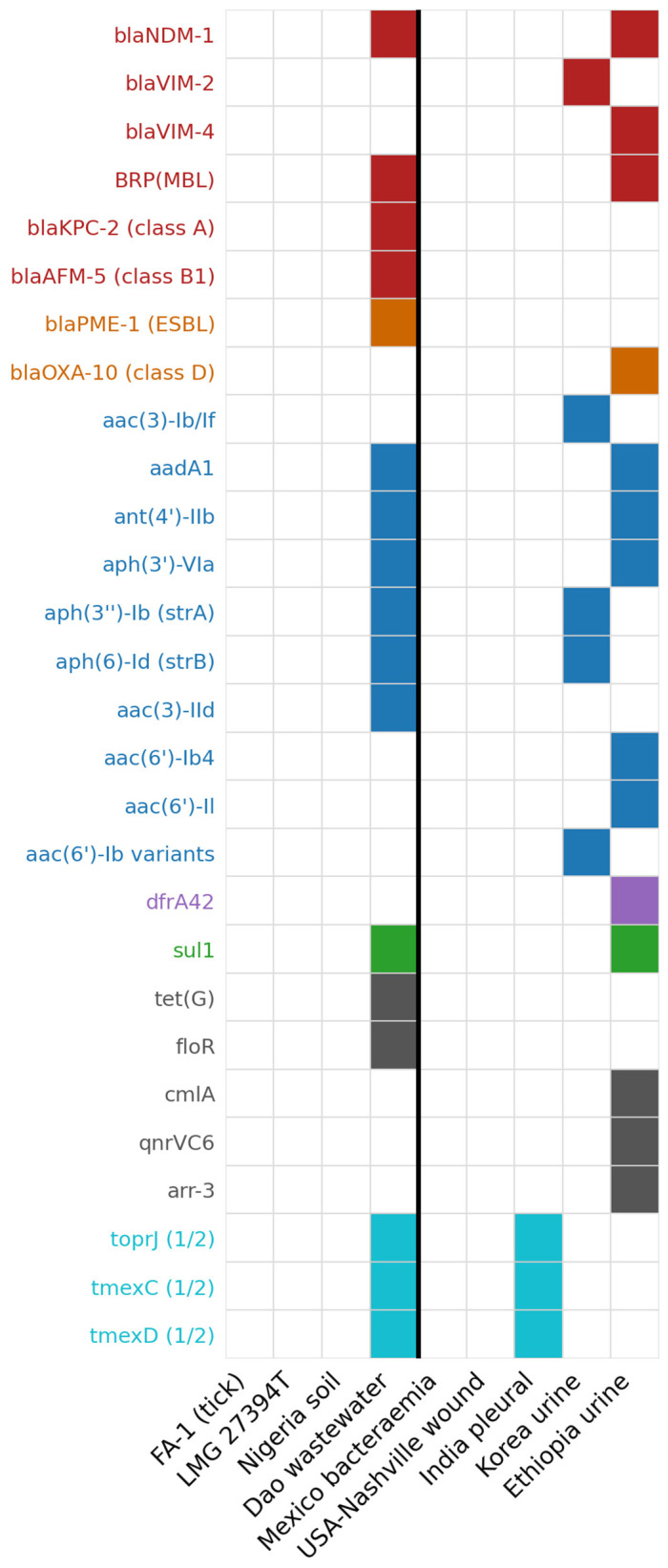
Consolidated acquired-resistance gene presence/absence heatmap across the nine-genome panel. Visualisation of the 28 unique acquired-AMR gene labels (rows) by isolate (columns) summarised in Table 2. Acquired-resistance carriage is concentrated in three MBL-positive isolates (with Dao additionally co-carrying the class A serine carbapenemase KPC-2): Dao Vietnam wastewater (17 genes including KPC-2, NDM-1, AFM-5, *strA*, *strB*, *aac(3)-IId*, and *aadA1*), Ethiopia clinical urine (14 genes anchored on two chromosomal NDM-1 cassettes plus plasmid VIM-4), and Korea clinical urine (five genes anchored on plasmid VIM-2). Of the remaining six genomes, India MGL_R1_GC carries three acquired-resistance genes (the *tmexCD-toprJ* tigecycline efflux operon); FA-1, type strain LMG 27394^T^, Nigeria ABJ_B2_1, Mexico GN-165 and USA-Nashville SAR321 carry zero acquired-resistance genes. A solid vertical black line separates the four environmental isolates (left: FA-1, LMG 27394^T^, Nigeria soil, Dao wastewater) from the five clinical isolates (right: Mexico blood culture, USA-Nashville wound, India pleural, Korea urine, Ethiopia urine). Abbreviations: AMR, antimicrobial resistance; MBL, metallo-β-lactamase. Cell colours follow the antibiotic-resistance-class scheme defined for Table 2: red, carbapenemases/metallo-β-lactamases; orange, other β-lactamases; blue, aminoglycosides; teal, tigecycline efflux; green, sulfonamide; purple, trimethoprim; grey, other classes.

**Figure 4:**
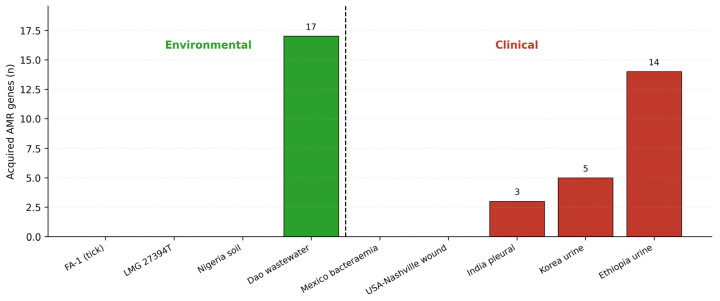
Per-genome acquired antimicrobial resistance burden. Bar plot of consolidated unique acquired-AMR gene counts per isolate, grouped by isolate stratum (environmental, left of dashed divider; clinical, right) with bars in ascending count order within each stratum. Carriage values: Dao (17) > Ethiopia (14) > Korea (5) > India (3); the five AMR-free genomes (FA-1, type strain LMG 27394^T^, ABJ_B2_1 Nigerian soil, Mexico GN-165, USA-Nashville SAR321) each carry 0 acquired-resistance genes. Bars coloured by isolate stratum (environmental = green; clinical = red). Abbreviations: AMR, antimicrobial resistance.

After removal of intrinsic *Pseudomonas* Mex-family efflux pumps and consolidation of database-source aliases (e.g., (Bla)*blaNDM-1* + NDM-1; (Bla)*blaVIM-2* + VIM-2; *aadA*/*aadA1*/(AGly)*aadA1*-pm/*ant(3″)-Ia* collapsed to a single Ethiopia-only locus) into canonical gene labels, 28 unique acquired-resistance genes were detected across the nine-genome dataset (with database-source aliases collapsed to canonical labels across CARD, NCBI AMRFinderPlus, ResFinder, and ARG-ANNOT; Figure 3, Table 2). The distribution was stratified between clinical and environmental compartments: one of four environmental genomes carried any acquired-resistance gene (the Dao Vietnam wastewater isolate carrying KPC-2 + NDM-1 + AFM-5 chromosomally; FA-1, the type strain, and the Nigerian soil isolate are AMR-free), compared with three of five clinical isolates (Figure 4). Per-genome consolidated unique-gene-label counts were Dao 17, Ethiopia 14, Korea 5, and India 3 (per-allele identity and coverage values, plus the full per-isolate gene lists across all four databases, are provided in Supplementary Table S5), summing to 39 isolate-gene label pairs across 28 unique acquired-resistance gene labels; 11 genes (NDM-1, *ble*-MBL, *ant(4′)-IIb*, *aph(3′)-VIa*, *aadA1*, *strA*, *strB*, *sul1*, *tmexC*, *tmexD*, and *toprJ*) were carried by more than one isolate, while every other acquired gene label was isolate-private. For Ethiopia, the count of 14 refers to unique acquired-resistance gene labels; because NDM-1 was detected at two physical loci (the 20.4 kb chromosomal cassette and a second copy ~2.9 Mb upstream), the isolate contains 15 physical acquired-AMR loci/copies after alias consolidation. Fisher’s exact testing on the directional hypothesis (clinical > environmental) under the expanded nine-genome panel did not reach conventional significance owing to the limited sample size (*n* = 9). For any acquired-AMR carriage, three of five clinical isolates and one of four environmental isolates were positive (two-tailed Fisher *p* = 0.524; one-tailed *p* = 0.357; odds ratio = 4.5; 95% CI = 0.25–80.5). For carbapenemase carriage, two of five clinical isolates and one of four environmental isolates were positive (two-tailed Fisher *p* = 1.000; one-tailed *p* = 0.595; odds ratio = 2.0; 95% CI = 0.11–35.8). These wide intervals reinforce that the stratum-level frequency contrasts are descriptive and underpowered, with two clinical isolates (Mexico and USA-Nashville) and three environmental isolates (FA-1, type strain, Nigerian soil) carrying no acquired-resistance genes.

Beyond presence/absence, the resistomes carried by the three resistant clinical isolates were mechanistically distinct rather than overlapping. HY1196, a Korean urine isolate from 2023, carried a VIM-2 carbapenemase together with a four-gene aminoglycoside resistance cluster after alias consolidation, *strA*/*aph(3″)-Ib*, *strB*/*aph(6)-Id*, *aac(3)-Ib/If*, and *aac(6′)-Ib* variants, a profile consistent with class 1 integron-borne resistance. MGL_R1_GC, an Indian pleural-fluid isolate from 2022, carried no β-lactamase but instead the three-component *tmexCD-toprJ* tigecycline efflux operon (*tmexC*, *tmexD*, *toprJ*; the *toprJ* locus produced two database aliases collapsed to a single canonical locus). PM99, an Ethiopian urine isolate from 2023, carried an exceptional 14-gene acquired-resistance profile after alias consolidation (25 raw database hit labels) anchored on two β-lactamases (NDM-1 and OXA-10) co-localised with a bleomycin resistance gene (*ble*-MBL/BRP(MBL)) on a single 20.4 kb chromosomal cassette, plus VIM-4 carried separately on a second replicon, accompanied by aminoglycoside (*aadA1*/*ant(3″)-Ia*, *ant(4′)-IIb*, *aph(3′)-VIa*, *aac(6′)-Ib4*, *aac(6′)-Il*), sulfonamide (*sul1*), trimethoprim (*dfrA42*), chloramphenicol (*cmlA* family, alleles *cmlA1*/*cmlA5*/*cmlA7* collapsed to a single canonical locus), rifamycin (*arr-3*), and fluoroquinolone (*qnrVC6*) resistance genes.

### 3.4. Mobile Genetic Element Burden and Mobility Typing Across the Nine-Genome Panel

Three orthogonal mobile-genetic-element (MGE) typing tools were applied to disentangle MGE-borne resistance architectures across the panel. MobileElementFinder returned stratified MGE counts: the three MBL-positive genomes carried 16–55 MGE hits (Dao 55; Ethiopia 22; Korea 16), markedly higher than the six MBL-negative genomes (range 0–8; *Pseudomonas guariconensis* type strain 2; FA-1 tick anchor 2; USA-Nashville 8; India 4; Mexico 0; Nigeria 0; ratio of medians 22/2 = 11-fold). Because raw MGE counts may scale with genome size, we also evaluated MGE density on a per-megabase basis using the assembly sizes in Table S1: the three MBL-positive genomes again ranked highest (Dao 8.7, Ethiopia 3.8, Korea 2.7 MGE/Mb), well above the 0–1.5 MGE/Mb spanned by the six MBL-negative genomes (median MGE density 3.8 vs. 0.4 MGE/Mb; ≈9.5-fold). Thus, the rank separation was retained after genome-size normalisation. MOB-suite relaxase typing on the five clinical or MBL-positive genomes returned MOBF + MOBH + MOBP relaxase-family hits for the Dao Vietnam wastewater chromosome (AP035765.1) and MOBF + MOBH hits for the Ethiopia chromosome (NZ_CP194068.1; chromosome sequence of assembly GCF_051550215.1 listed in Table 1). The Dao and Ethiopia chromosomes shared a MOBH-family relaxase signal. IntegronFinder discriminated two distinct integron architectures: the Ethiopia clinical genome carried six integron structures (two complete chromosomal integrons, one CALIN cluster and one In0 element on the chromosome, plus one complete integron and one CALIN cluster on plasmid NZ_CP194069.1), consistent with classical class 1 integron-mediated multi-class resistance; in contrast, no classical integron structures were detected on the Dao chromosome despite the presence of NDM-1, KPC-2 and AFM-5, with a single complete integron found only on the Dao plasmid pKNHN1 (AP035766.1), separate from the chromosomal carbapenemase loci. The Dao chromosomal multi-resistance cassettes were classified as IS-element-bounded transposon-mediated resistance islands rather than classical class 1 integrons. The Korea genome carried CALIN and In0 elements adjacent to its VIM-2 contig, consistent with degraded integron architecture, and the remaining six genomes were integron-negative or carried empty In0 structures.

### 3.5. The Ethiopia Chromosomal NDM-1 + OXA-10 Cassette Is a Mobile Genetic Element Absent from MBL-Negative Environmental Comparators

To test the mobile-element acquisition hypothesis at sequence resolution, we extracted the genomic context of every metallo-β-lactamase and OXA-family β-lactamase hit in the clinical genomes (±10 kb flanking) and aligned each to the three environmental comparators with minimap2. The most informative locus was the Ethiopia chromosomal cassette spanning NZ_CP194068.1:5,739,230–5,759,654 (20.4 kb), which contains, within a single contiguous interval, OXA-10 (NZ_CP194068.1:5,749,230–5,750,030), the bleomycin resistance gene BRP(MBL) (NCBI: *ble*-MBL; 5,753,659–5,754,024), and NDM-1 (5,754,028–5,754,840), interspersed with 33 additional CDS predicted by Prodigal. The same Ethiopia genome carried two additional acquired β-lactamases outside this cassette window: a VIM-4 gene on a separate replicon (NZ_CP194069.1:68,142–68,942) and a second NDM-1 paralogous copy located ~2.9 Mb upstream on the same chromosome (NZ_CP194068.1:2,831,950–2,832,762); both lie outside the 20.4 kb cassette analysed here.

When the entire 20.4 kb Ethiopia cassette region was aligned against each MBL-negative environmental-comparator genome, FA-1 produced no minimap2 alignment whatsoever (0% query coverage), while the type strain LMG 27394^T^ and the Nigerian soil isolate ABJ_B2_1 each produced a single high-confidence alignment block of 3.7 kb and 3.6 kb, respectively (18.2% and 17.8% query coverage, MAPQ = 60). Both environmental syntenic blocks aligned to the same narrow segment of the Ethiopia query, qstart 279–3991 (forward strand) and 279–3923 (reverse-complement strand), respectively, corresponding to the leftmost ~4 kb of the 20.4 kb window. The remaining ~16–17 kb of the Ethiopia cassette, which contains the entire NDM-1 + OXA-10 + BRP(MBL) gene cluster and the surrounding mobile-element machinery, has no homologous sequence in any of the three MBL-negative environmental-comparator genomes. The independent convergence of two environmental genomes on the same alignment-junction position (qstart = 279) suggests this is a defined insertion-site boundary at which the cassette was acquired into the chromosome of the Ethiopia ancestor, leaving the conserved bacterial backbone immediately flanking the insertion site shared with the environmental clade, but the cassette itself entirely foreign.

**Figure 5:**
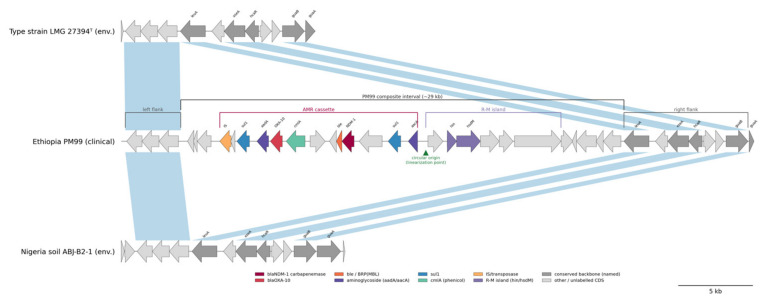
Gene-level synteny of the Ethiopia PM99 NDM-1/OXA-10 region across the circular chromosomal origin. The Ethiopia PM99 chromosome (NZ_CP194068.1, circular) is linearised across its origin and reads left to right: conserved left-flank backbone → AMR cassette (including IS6100, *sul1*, *aadA*, OXA-10, *cmlA*, *ble*/BRP(MBL), NDM-1, *sul1*, *aacA*) → restriction–modification (R-M) island (*hin*, *hsdM*) → conserved right-flank backbone (*leuA*, *xseA*, *hcaR*, *guaB*). The circular-origin linearisation point (between the cassette and the R-M island) is a display feature, not a biological junction. Arrows indicate annotated gene orientation. In the *P. guariconensis* type strain LMG 27394^T^ and the Nigerian soil isolate ABJ_B2_1, the same left- and right-flank backbone genes are directly adjacent, so the ~29 kb PM99 composite interval (AMR cassette plus the adjacent R-M island) is absent from the corresponding syntenic backbone of both comparators; the tick-derived FA-1 anchor produced no detectable alignment at this locus. Blue ribbons are gene-level synteny links between shared annotated backbone genes, not nucleotide-identity ribbons; interval boundaries are approximate and inferred from annotated gene order rather than contiguous nucleotide alignment across the junctions. The Nigeria track is reverse complemented for display. Because the comparator genomes are draft assemblies, absence at this locus does not exclude related sequences in unassembled regions.

Because the Ethiopia PM99 chromosome (NZ_CP194068.1) is a closed circular replicon, the cassette window above reached the record origin rather than a true terminus. Extending the comparison across this origin at the gene level showed the conserved backbone resuming on the far side of the cassette: a restriction–modification island (*hin*, *hsdM*) followed by four conserved genes (*leuA*, *xseA*, *hcaR*, *guaB*) whose orthologues, in both the type strain LMG 27394^T^ and the Nigerian soil isolate, sat directly adjacent to the shared left-flank backbone with no intervening sequence. The AMR cassette and the adjacent R-M island thus together formed a ~29 kb interval that interrupted an otherwise conserved backbone locus, absent from the corresponding syntenic region of both comparators, consistent with a composite chromosomal insertion in PM99 bracketed by conserved backbone on both sides (Figure 5). Boundaries are approximate and inferred from annotated gene order; because the comparator assemblies are drafts, absence here does not exclude related sequences in unassembled regions.

### 3.6. The Ethiopia Chromosome Carries Two Distinct NDM-1 Mobility Architectures

To distinguish whether the Section 3.5 Ethiopia chromosomal cassette functions as an autonomous integrative and conjugative element (ICE) or as a non-autonomously mobilisable insertion-bounded element, the cassette region and the full Ethiopia chromosome were analysed with ICEfinder. Two positive controls returned expected positive-control calls: the ICEfinder built-in reference *Klebsiella pneumoniae* HS11286 (CP003200.1, 5.33 Mb; Job ID VCFVX3Y5uo), which returned two T4SS-type ICEs of 62.2 kb and 59.1 kb at expected coordinates; and the canonical *Pseudomonas* ICE region of *Pseudomonas putida* B13 (ICEclc, GenBank AJ617740, 105 kb; Job ID ScJjdE558k), which returned one T4SS-associated module of 24.1 kb (50,246–74,302) corresponding to the core conjugation region of ICEclc, returning the expected positive-control call for *Pseudomonas* ICE architecture under default parameters.

ICEfinder analysis of the extracted Section 3.5 cassette sequence (NZ_CP194068.1:5,740,000 to the chromosome end at 5,759,654, ~19.6 kb of the 20.4 kb interval; Job ID lcq5duHdss) returned no complete T4SS-type ICE prediction. ICEfinder therefore did not detect a contiguous integrase/relaxase/T4CP/VirB4-type module set within the cassette sufficient for a complete ICE call, consistent with the IS- and transposase-associated flanking machinery described in Section 3.5 but with no autonomous T4SS-encoding module embedded in the cassette itself. The full-chromosome scan of the same Ethiopia replicon (NZ_CP194068.1, 5,759,654 bp; Job ID xQ52e0OWUk) returned one complete T4SS-type ICE prediction under default parameters, spanning 2,880,460–2,904,589 (24,130 bp), located approximately 48 kb downstream of the second Ethiopia NDM-1 copy at 2,831,950–2,832,762 reported in Section 3.5. This was the only complete ICE prediction returned by ICEfinder on the Ethiopia chromosome.

The Ethiopia chromosome therefore carried two distinct NDM-1 mobility architectures within the same replicon. The upstream NDM-1 copy at ~2.83 Mb sat within ~50 kb of an ICEfinder-predicted T4SS-encoding ICE, indicating an ICE-associated genomic neighbourhood. The Section 3.5 multi-resistance cassette at ~5.75 Mb, which carried NDM-1 + OXA-10 + BRP(MBL) and is absent from environmental comparators, is not adjacent to an ICEfinder-predicted ICE on this chromosome: the IS- and transposase-bounded architecture supports historical insertion-mediated acquisition rather than ongoing autonomous self-conjugation. The cassette sequence also showed a markedly lower GC content (56.8%) than the surrounding chromosome backbone (62.1%). We note these as in silico positional and compositional observations; functional conjugation assays and long-read structural characterisation are needed to establish the present-day mobility of either NDM-1 architecture. ICEfinder Job IDs are listed in Supplementary Table S4 for reproducibility.

### 3.7. Adjacent ICE Architecture Co-Localises with IS-Bounded AMR Cassettes Across the Panel

To extend the ICEfinder analysis described in Section 3.6 to the full nine-genome panel, full-chromosome ICEfinder scans were submitted for the two MBL-positive chromosomal genomes carrying the heaviest carbapenemase load, the Dao Vietnam wastewater chromosome AP035765.1 (Job ID rrVozGYfMQ, 5,883,648 bp) and the Ethiopia urine chromosome NZ_CP194068.1 (Job ID xQ52e0OWUk, 5,759,654 bp). The Dao chromosomal scan returned four complete T4SS-type ICE predictions spanning 44.4 kb, 101.4 kb, 24.1 kb and 31.5 kb under default parameters. Three of the four Dao ICEs co-localised within 27–50 kb of a chromosomal carbapenemase hotspot identified by ABRicate: an ICE of 44.4 kb at 1,583,317–1,627,695 located ~45 kb downstream of the KPC-2 locus (1,537,989–1,538,870); an ICE of 24.1 kb at 3,698,093–3,722,222 located ~27 kb downstream of the NDM-1 locus (3,671,399–3,672,211); and an ICE of 31.5 kb at 4,641,556–4,673,032 located ~31 kb downstream of the AFM-5 locus (4,610,546–4,611,349). The fourth Dao ICE (101.4 kb at 2,118,851–2,220,211) is a standalone element with no adjacent ABRicate-detected carbapenemase. The Ethiopia chromosomal scan returned one complete T4SS-type ICE prediction of 24.1 kb at 2,880,460–2,904,589, located ~48 kb downstream of the upstream Ethiopia NDM-1 copy (2,831,950–2,832,762; Section 3.6). The cassettes themselves, described in Section 3.5 and Section 3.6, lack contiguous T4SS-encoding modules within their immediate 25 kb windows; the autonomous conjugation machinery sat in adjacent but distinct genomic neighbourhoods. All ICEfinder Job IDs are listed in Supplementary Table S4 for reproducibility. An independent oriTfinder2 analysis of these two chromosomes returned a complete conjugative region on the Dao chromosome, a predicted oriT (~1.60 Mb; oriTDB H-value 0.82), a MOBF-family (TraI_2) relaxase, a T4CP, and a T4SS gene cluster co-localised at ~1.60–1.63 Mb, whereas the Ethiopia chromosome carried the conjugation machinery (a MOBF-family relaxase, a T4CP, and a T4SS gene cluster at ~2.88–2.90 Mb) without a detectable oriT (Figure S2).

## 4. Discussion

Comparative phylogenomic analysis of nine *Pseudomonas guariconensis*-clade genomes, four environmental and five clinical, spanning four continents and a tick-vector environmental anchor, supports a model in which the recently acquired resistance carriage observed in some *P. guariconensis* clinical isolates is associated with mobile-element acquisition of resistance determinants [44] rather than with features intrinsic to the lineage. Three lines of evidence converge on this conclusion. First, the acquired-AMR distribution separates the MBL-negative environmental comparators from the MGE-rich resistant genomes: FA-1, the LMG 27394^T^ type strain, and the Nigerian soil isolate carry no acquired-resistance genes in our uniform screen, whereas the Dao Vietnam wastewater isolate carries chromosomally integrated carbapenemases, and three of five clinical genomes carry between three and 14 consolidated acquired-resistance genes. Second, the resistomes of the three resistant clinical isolates are mechanistically distinct (HY1196 VIM-2 + aminoglycoside cluster; MGL_R1_GC *tmexCD-toprJ* tigecycline efflux; PM99 chromosomal NDM-1 + OXA-10 cassette plus a separate-replicon VIM-4), excluding clonal dissemination as a parsimonious explanation and instead supporting independent horizontal acquisition events from three different mobile-element sources. Third, sequence-resolution synteny analysis of the most striking clinical resistance locus, the Ethiopia chromosomal NDM-1 + OXA-10 cassette, confirmed that more than 80% of the cassette region, including the entire NDM-1 + OXA-10 + *ble*-MBL gene cluster, is absent from the three MBL-negative environmental-comparator genomes, with the conserved bacterial backbone flanking a defined insertion-site boundary (qstart ≈ 279) shared by the type strain and Nigerian soil genomes (FA-1 produced no detectable alignment to the cassette region at the alignment threshold used, consistent with its deeper ANI divergence from the panel, and is therefore not informative as an absence call). The convergence of two environmental genomes on the same junction position provides additional evidence consistent with a defined MGE insertion site rather than an alignment artefact. Throughout this study, ‘MBL-positive’ refers to an in silico ABRicate consensus classification (≥80% identity/≥80% coverage across ≥2 of the four ABRicate AMR databases); these calls are genotypic predictions and have not been validated by phenotypic susceptibility testing. Because the panel comprises only nine genomes and includes a resistant wastewater isolate, the clinical-versus-environmental frequency contrast is statistically underpowered and does not reach conventional significance (Section 3.3). We therefore treat the distributional pattern as descriptive and exploratory, with the mobile-element-acquisition conclusion resting primarily on sequence-resolution synteny and MGE-context evidence rather than on stratum-level frequencies. The India *tmexCD-toprJ* profile resembles the plasmid-borne *tmexCD1*-*toprJ1* acquisitions reported previously in *Enterobacteriaceae* and *Pseudomonas*, including the original animal-origin *K. pneumoniae* report [45].

The FA-1 environmental anchor adds two new dimensions to the comparative reference set for this clade. First, taxonomically, FA-1 sits at 87.5–87.9% ANI to all other clade members, well below the conventional 95–96% species threshold, and is recovered on the longest external branch of the core-genome phylogeny with maximal bootstrap support, consistent with placement outside *P. guariconensis* sensu stricto; the current NCBI type-strain ANI taxonomy check places FA-1 with the recently described *Pseudomonas shiyinii* (Section 3.1). Because FA-1 is conspecific with the already described *Pseudomonas shiyinii* (Section 3.1), no new species description is warranted; we frame FA-1 as a *P. shiyinii*-related environmental anchor for ongoing comparative analyses, with the underlying genome publicly released under accession JBWXTH010000000. Second, ecologically, FA-1, a *P. shiyinii*-related isolate recovered from a camel tick, broadens the known ecological context of this lineage, whose type strain (ST4) derives from a vegetable rhizosphere. The internal tissues of *Hyalomma dromedarii*, a tick species ecologically associated with camels across the Arabian Peninsula and East Africa, provide a plausible reservoir-level environmental compartment in which the clade may circulate without acquired resistance, providing context for future surveillance of AMR exchange between environmental and clinical compartments.

HY1196 also requires taxonomic caution, sitting at 91.61–91.87% ANI to every other clade member except the more divergent FA-1 anchor (~87.9%), including the type strain, a position that is below the species threshold (~95%) but above conventional genus-level boundaries (~80%) and consistent with cryptic sub-species structure within currently labelled *P. guariconensis*. HY1196’s isolated long branch in the core-genome phylogeny (0.0709), outside the tight species-level cluster but within the broader clade, mirrors its FastANI position and reinforces its borderline intra-clade/candidate sister-genomospecies designation. Because NCBI taxonomic assignment is conservative and frequently lags behind ANI-based reclassification at the borderline range, we interpret this finding as a flag for future taxonomic revision of the clade rather than as a contradiction of the central hypothesis.

PM99 stands out because its 14-gene acquired-resistance profile (post alias consolidation; 25 raw database hit labels) and chromosomal NDM-1 + OXA-10 cassette are without precedent in *P. guariconensis*: the prior literature reports single-carbapenemase acquisitions on plasmids or integrative conjugative elements [46] (Tunisia OXA-204 [2]; Vietnam wastewater NDM-1 + KPC-2 on ICEs [6]; Brazil BIM-1 [47]), but to our knowledge no prior *P. guariconensis* isolate has been reported with co-localised NDM-1 + OXA-10 + *ble*-MBL on a single ~20 kb chromosomal cassette accompanied by a separate-replicon VIM-4. Whether this represents a single integrated mobile-element acquisition event or a stepwise accretion of distinct resistance modules at the same chromosomal locus cannot be resolved from a single isolate genome and is a priority for follow-up sequencing of additional Ethiopian and East African *P. guariconensis clinical* isolates.

The ICEfinder analysis reported in Section 3.6 refines but does not replace the mobile-element acquisition hypothesis. Two interpretations of the Ethiopia chromosome are now distinguishable. The upstream NDM-1 copy at ~2.83 Mb resides in an ICE-associated neighbourhood, with a complete ICEfinder-predicted T4SS-encoding ICE within ~50 kb; this is the prototypical architecture of ICE-encoded conjugation modules described in Gram-negative pathogens [46]. The Section 3.5 multi-resistance cassette at ~5.75 Mb has a different signature: the cassette carries the heavier resistance cargo (NDM-1 + OXA-10 + BRP(MBL) plus the surrounding integrase- and transposase-flanked machinery described in Section 3.5), yet lacks an adjacent autonomous ICE on the same replicon and shows a markedly lower GC content (56.8% vs. 62.1%) characteristic of horizontal acquisition from a donor of distinct base composition. We interpret this contrast as evidence that the two NDM-1 loci reached their present chromosomal position by mechanistically distinct routes, with the multi-resistance cassette compatible with historical insertion- or plasmid-mediated acquisition followed by chromosomal fixation rather than ongoing autonomous self-conjugation. This refined interpretation does not require revisiting the synteny evidence presented in Section 3.5: both the absence of the cassette from environmental comparators and the defined insertion-site boundary shared with environmental backbone genomes (Section 3.5) fit the historical-acquisition model.

The expanded full-chromosome ICEfinder analysis described in Section 3.7 adds an architectural layer to this interpretation. These ICEfinder predictions are supported by hallmark conjugation-associated features already detected in the same genomes: MOB-suite identified chromosomal MOB-family relaxase signals in Dao (MOBF/MOBH/MOBP) and Ethiopia (MOBF/MOBH), while the ICEfinder complete-ICE calls corresponded to T4SS-encoding modules; together, these features support conjugation-associated architecture, although transfer of the specific cassettes was not functionally demonstrated. An independent conjugation-module predictor, oriTfinder2 [34], further corroborated this architecture: it identified a complete conjugative region (oriT, relaxase, T4CP and T4SS) on the Dao chromosome and the conjugation machinery without a detectable oriT on the Ethiopia chromosome, consistent with the Ethiopia cassette behaving as mobilisable cargo rather than a fully autonomous self-transmissible element. Across the panel, every IS-element-bounded chromosomal carbapenemase cassette in the two heaviest MBL-positive genomes (Dao chromosomal cassettes at KPC-2, NDM-1, and AFM-5; Ethiopia upstream NDM-1 copy at ~2.83 Mb) sits within 27–50 kb of an independent T4SS-encoding ICE on the same replicon, but no carbapenemase gene is itself contained within an ICEfinder-predicted complete T4SS module. The 50 kb window is used here as a descriptive proximity threshold for visualising potential cis-supplied conjugation neighbourhoods; we do not claim it as a mechanistically validated cutoff, and ICE-cassette mobility was not functionally tested in this study. We interpret this co-localisation as architectural rather than mechanistic: the data are consistent with the IS-bounded cassettes acting as mobilisable cargo whose conjugation could be supplied in cis by the adjacent autonomous ICEs, but conjugation of these specific cassettes has not been functionally demonstrated. The Dao et al. paper experimentally validated conjugation of its NDM-1- and KPC-2-bearing ICE regions to *Pseudomonas putida* KT2440 at ~10^−2^ frequency [6]; whether the IS-bounded cassettes themselves transfer at comparable rates when their adjacent ICEs are active is a priority for future mating-out experiments. The shared MOBH-family relaxase signal between Dao and Ethiopia (Section 3.4), the convergent IS-bounded cassette/adjacent autonomous-ICE architecture observed across MBL-positive chromosomal genomes (Section 3.5, Section 3.6 and Section 3.7), and the absence of any acquired-resistance gene from the three integron-free environmental genomes (FA-1, type strain, Nigerian soil; Section 3.3) are jointly consistent with an architectural One Health hypothesis (without demonstrating directionality or transmission) in which mobile-element acquisition of carbapenemase determinants is observable in both wastewater and clinical compartments, mediated by architecturally convergent but mechanistically distinct strategies in the two compartments.

The evolutionary relevance of the shared MOBH signal is that relaxases mark the conjugation module rather than the resistance cargo. Relaxases initiate conjugative DNA transfer, and MOB families are used as phylogenetic classes of mobilisation systems [48]. Thus, the shared MOBH-family signal in the Dao wastewater and Ethiopia clinical chromosomes is consistent with related mobilisation machinery occurring across environmental-interface and clinical compartments of the clade. The Dao isolate provides a direct *P. guariconensis* precedent for MOBH-type relaxase-bearing ICEs carrying carbapenemase genes [6], but we did not identify evidence that the complete Dao and Ethiopia resistance-cassette architectures are identical or widely described elsewhere in *Pseudomonas*. We therefore interpret the shared MOBH marker cautiously, as support for related conjugation-module ancestry or exchange potential, not as proof of transfer of the same complete resistance cassette; functional conjugation of the Ethiopia element was not demonstrated.

From a One Health perspective, the Dao Vietnam wastewater isolate is the most notable environmental-interface genome in the panel—a wastewater-derived *P. guariconensis* carrying clinically important carbapenemases (KPC-2, NDM-1 and AFM-5) on the chromosome and the heaviest acquired-resistance burden of any genome examined here. Wastewater is not a pristine environmental compartment, but a convergence point for hospital effluent, community sewage and environmental inputs; therefore, the carbapenemase determinants in Dao cannot be assigned to a single origin. Rather than proving a standalone environmental source, this finding shows that high-consequence carbapenem-resistance genes are present at the environmental–clinical interface of the clade, consistent with wastewater acting as a reservoir and mixing point for mobile resistance genes. Because the dataset is cross-sectional, we cannot infer the direction of exchange between wastewater and clinical compartments.

Our analysis has several limitations. The total sample size (*n* = 9 genomes) limits statistical power for Fisher’s exact testing of the resistance-distribution contrast; although the directional pattern (3/5 clinical vs. 1/4 environmental for any acquired-resistance gene; 2/5 clinical vs. 1/4 environmental for carbapenemases, the single MBL-positive environmental genome being the Dao et al. Vietnam wastewater isolate that nonetheless groups within the clinical sensu stricto clade) is consistent across the dataset, none of the cross-stratum tests reaches conventional significance under conservative two-tailed Fisher’s testing, and the mechanistic conclusions therefore rest on the sequence-resolution synteny evidence (Section 3.5) rather than on the resistance-frequency contrast alone. Both odds-ratio confidence intervals span the null value of one, so these point estimates should be read as indicating only the direction of the observed pattern rather than reliable effect-size estimates. Public-genome availability for environmental *P. guariconensis* isolates remains a constraint: the Nigerian soil genome, the original Venezuelan type strain, the FA-1 tick-vector environmental anchor reported here, and the recently published Dao et al. Vietnam wastewater isolate together exhaust the publicly available environmental reference set used in this study. Environmental *P. guariconensis* isolates carrying acquired metallo-β-lactamases on integrative conjugative elements have been reported in wastewater [6]; the MBL-negative pattern observed here therefore applies to the three specific environmental genomes analysed and not to the species’ global environmental reservoir. Expanding the environmental reference set is a clear priority for future work. Contamination of the FA-1 assembly was assessed by BUSCO completeness (99.2% complete, 0.0% missing, Section 2.2), by CheckM2 v1.1.0 (100.0% completeness, 0.1% contamination, Neural Network specific model), and by the upstream Kraken2 taxonomic classification of the source reads (96.0% of classified reads assigned to *Pseudomonas*); the assembly is therefore essentially contamination-free at the limits of current ML-based prediction. We did not run GTDB-Tk separately; placement relative to *P. guariconensis* was supported by all-vs-all FastANI within the nine-genome panel, the core-genome ML phylogeny, and dDDH, while FA-1’s reassignment as a *Pseudomonas shiyinii*-related isolate rests on the current NCBI type-strain ANI taxonomy check (Section 3.1). Second, isolate-specific phenotypic susceptibility testing was not available for FA-1 here, so direct minimum inhibitory concentration testing of the new environmental anchor could not be performed; therefore, we frame the genomic AMR profile as exhibiting collection-level concordance with the upstream survey [10], rather than per-isolate validation. More broadly, all resistance interpretations in this study, for FA-1 and for the public comparator genomes alike, should be understood as genomic determinant carriage rather than confirmed phenotypic resistance or susceptibility: gene presence does not necessarily indicate expression or clinical resistance, and absence of acquired-resistance genes does not exclude resistance mediated by regulatory changes, efflux activity, porin variation, or point mutations not captured by acquired-gene screening. Third, transposase and integrase identification in the Ethiopia cassette-flanking region is by Prodigal CDS prediction inference; formal HMMER profile annotation against the ISFinder and INTEGRALL databases would refine the mobile-element classification in a follow-up study. Fourth, because the genomes analysed here are short-read draft assemblies, the exact contiguity of repetitive regions, insertion sequences, ICE boundaries, and larger structural rearrangements should be interpreted cautiously; long-read or hybrid sequencing using Oxford Nanopore or PacBio platforms would improve assembly continuity and provide stronger resolution of insertion boundaries, cassette organisation, and structural rearrangements, so that this analysis supports gene-level and scaffold-level MGE comparisons while complete structural resolution remains a target for future work.

## 5. Conclusions

Comparative phylogenomic analysis of nine *P. guariconensis*-clade genomes anchored on a tick-vector environmental isolate (FA-1) supports a horizontal-acquisition model in this dataset: the three MBL-negative environmental comparators (FA-1, LMG 27394^T^, and ABJ_B2_1) carry no acquired AMR in our uniform screen, whereas the Dao Vietnam wastewater isolate and three resistant clinical isolates carry mechanistically distinct mobile-element-associated resistomes. PM99 is an informative example, with a chromosomal NDM-1 + OXA-10 cassette accompanied by a separate-replicon VIM-4; more than 80% of the 20.4 kb cassette region is absent from the MBL-negative environmental comparators, with a defined insertion-site boundary shared by the type strain and Nigerian soil genomes. Continued genomic surveillance of arthropod-vector, wastewater and clinical *P. guariconensis* and related lineages is needed to monitor the trajectory of resistance acquisition in this emerging opportunistic pathogen.

## Figures and Tables

**Table 1:** Nine *P. guariconensis*-clade genomes analysed in this study. GN-165 was recovered from a venipuncture blood culture; ‘bacteraemia’ in Figures 2–4 is used as clinical shorthand for this blood-culture isolate.

Strain	Country	Source	Stratum	Year	Assembly Accession	Assembly Level
FA-1 (this study)	Saudi Arabia/Hail	*Hyalomma dromedarii* internal tissues	Environmental	2024	JBWXTH000000000 (WGS master; version JBWXTH010000000)	Draft WGS (38 contigs; N50 363 kb)
LMG 27394^T^	Venezuela	*Vigna unguiculata* rhizosphere soil	Environmental (type strain)	2013	GCF_900102675.1	Scaffold (N50 313 kb)
ABJ_B2_1	Nigeria	Soil	Environmental	2024	GCF_044903045.1	Contig (N50 262 kb)
HY1196	South Korea	Urine	Clinical	2023	GCF_034724015.1	Scaffold (N50 322 kb; scaffold N50 376 kb)
MGL_R1_GC	India/Chhattisgarh	Pleural fluid	Clinical	2022	GCF_036528595.1	Scaffold (N50 322 kb; scaffold N50 441 kb)
GN-165	Mexico/Nuevo Leon	Blood culture	Clinical	2022	GCF_036879405.1	Contig (N50 331 kb)
PM99	Ethiopia/Addis Ababa	Urine	Clinical	2023	GCF_051550215.1	Complete genome (closed)
SAR321	USA/Nashville, TN	Wound	Clinical	2025	GCF_052077635.1	Contig (1 contig, 5.23 Mb)
KNHN1 (Dao et al.)	Vietnam/Hanoi	Urban wastewater (hospital-adjacent)	Environmental, MBL+	2021	AP035765 chromosome + AP035766 plasmid (DDBJ)	Complete (chromosome + plasmid, both circular)

**Table 2:** Consolidated acquired-resistance determinant matrix across the full nine-genome panel. Dao et al. [6] Vietnam wastewater isolate KNHN1 is included in the matrix; the per-allele ABRicate consensus for all nine genomes is reported in Supplementary Table S5. Abbreviations: AMR, antimicrobial resistance; MBL, metallo-β-lactamase. Resistance-gene databases (CARD, NCBI AMRFinderPlus, ResFinder, ARG-ANNOT) are detailed in Section 2.6. Coloured cells indicate determinant presence; blank cells indicate absence.

Determinant	FA-1	LMG 27394^T^	ABJ_B2_1	HY1196	MGL_R1_GC	GN-165	PM99	SAR321	KNHN1
NDM-1									
KPC-2									
AFM-5									
PME-1									
*tet(G)*									
*floR*									
VIM-2									
VIM-4									
OXA-10									
*ble*-MBL									
*aac(3)-Ib/If*									
*aac(3)-IId*									
*aac(6′)-Ib* variants									
*aac(6′)-Ib4*									
*aac(6′)-Il*									
*ant(4′)-IIb*									
*aph(3′)-VIa*									
*strA*/*aph(3″)-Ib*									
*strB*/*aph(6)-Id*									
*aadA1*/*ant(3″)-Ia*									
*tmexC*									
*tmexD*									
*toprJ*									
*dfrA42*									
*sul1*									
*cmlA*									
*qnrVC6*									
*arr-3*									
Total acquired (consolidated)	0	0	0	5	3	0	14	0	17

Cell shading denotes the antibiotic-resistance class of each detected determinant: red, carbapenemases/metallo-β-lactamases and the associated *ble*-MBL cassette determinant (NDM-1, KPC-2, AFM-5, VIM-2, VIM-4, *ble*-MBL); orange, other β-lactamases (OXA-10, PME-1); blue, aminoglycoside-modifying enzymes; teal, tigecycline efflux (*tmexCD-toprJ*); green, sulfonamide (*sul1*); purple, trimethoprim (*dfrA42*); grey, other classes (rifamycin, phenicol, quinolone and tetracycline).

## Data Availability

This Whole Genome Shotgun project has been deposited at DDBJ/ENA/GenBank under the accession JBWXTH000000000. The version described in this paper is version JBWXTH010000000. Raw reads: SRR38121108 (full WGS) and SRR38121107 (purity check). BioProject: PRJNA1449514; BioSample: SAMN57124393. Additional intermediate result files (FastANI matrices, IQ-TREE tree outputs, Panaroo gene-presence–absence tables, ABRicate raw outputs, and minimap2 PAFs) are available from the corresponding authors upon reasonable request.

## Supplementary Materials

The following supporting information can be downloaded at: https://www.mdpi.com/article/10.3390/microorganisms14071428/s1, Figure S1: Genome BLAST Distance Phylogeny (GBDP) tree placing FA-1 among the closest *Pseudomonas* type strains identified by TYGS. Figure S2: Conjugation-module maps (oriT, relaxase, T4CP and T4SS) of the Dao and Ethiopia chromosomes predicted by oriTfinder2. Table S1: Reference genomes used in FastANI and phylogenomic analyses. Table S2: Raw CARD database hits from ABRicate v1.4.0 (first-pass screen; all hits shown for transparency, including those below the consensus thresholds). Table S3: Negative-database summary (ResFinder + NCBI AMR reference set), no acquired-resistance genes detected in the environmental MBL-negative trio. Table S4: ICEfinder analysis Job IDs and results for all submissions performed in this study (ICEberg database). Table S5: Per-allele acquired antimicrobial-resistance-gene consensus across the nine-genome *Pseudomonas guariconensis*-clade panel (ABRicate v1.4.0 against CARD, NCBI AMRFinderPlus, ResFinder, and ARG-ANNOT databases; PlasmidFinder was run separately for plasmid-replicon screening and not included in the AMR consensus). Table S6: Pairwise average amino-acid identity (AAI) matrix across the nine-genome clade panel (mean identity over 3197 single-copy core orthologues). File S1: Pre-WGS culture-purity check, taxonomic profiling of preliminary shotgun dataset (SRA SRR38121107) by Kraken2 v2.1 and MetaPhlAn4.

## Abbreviations

The following abbreviations are used in this manuscript:

AMR	Antimicrobial Resistance
ANI	Average Nucleotide Identity
ARG	Antibiotic Resistance Gene
BRP	Bleomycin Resistance Protein
BUSCO	Benchmarking Universal Single-Copy Orthologs
CALIN	Cluster of attC sites Lacking Integron-integrase
CARD	Comprehensive Antibiotic Resistance Database
CDS	Coding DNA Sequence
CTAB	Cetyltrimethylammonium Bromide
DDBJ	DNA Data Bank of Japan
ENA	European Nucleotide Archive
GC	Guanine–Cytosine
ICE	Integrative and Conjugative Element
In0	Integron with integrase but no gene cassettes
IRB	Institutional Review Board
IS	Insertion Sequence
KPC	*Klebsiella pneumoniae* Carbapenemase
LCA	Lowest Common Ancestor
MBL	Metallo-β-Lactamase
MGE	Mobile Genetic Element
MIC	Minimum Inhibitory Concentration
ML	Maximum Likelihood
MOBF	Mobilisation F (relaxase family)
MOBH	Mobilisation H (relaxase family)
MOBP	Mobilisation P (relaxase family)
NCBI	National Center for Biotechnology Information
NDM	New Delhi Metallo-β-lactamase
OXA	Oxacillinase
SH-aLRT	Shimodaira–Hasegawa approximate Likelihood Ratio Test
SRA	Sequence Read Archive
T4CP	Type IV Coupling Protein
T4SS	Type IV Secretion System
UFBoot	Ultrafast Bootstrap
VIM	Verona Integron-encoded Metallo-β-lactamase
WGS	Whole-Genome Sequencing
