# Supplementary material for: Genomic Evidence for Mobile-Element-Associated Resistance: Predicted MOBH-Family Relaxase Sharing and Adjacent ICE-Cassette Architectures in the Pseudomonas guariconensis Clade
Source: Microorganisms. 2026 Jun 30;14(7):1428. doi: 10.3390/microorganisms14071428 (PMC13413775; doi:10.3390/microorganisms14071428)
Supplement: Supplementary file 1 [file microorganisms-14-01428-s001.zip › microorganisms-4380008-supplementary.pdf]

Supplementary Materials

Genomic Evidence for Mobile-Element-Associated Resistance: Predicted MOBH-Family Relaxase Sharing and Adjacent ICE-Cassette Architectures in the *Pseudomonas guariconensis* Clade

Fuad Alanazi, Abdulhadi M. Abdulwahed, Abdulrahman Alrezaihi, Mohammed Ali M. Marie, Alanoud T. Aljasham, Raed Farzan

Contents

Figure S1. Genome BLAST Distance Phylogeny (GBDP) tree placing FA-1 among the closest *Pseudomonas* type strains identified by TYGS.

Figure S2. Conjugation-module maps (oriTfinder2) of the Dao and Ethiopia chromosomes.

Table S1. Reference genomes used in FastANI and phylogenomic analyses.

Table S2. Raw CARD database hits from ABRicate v1.4.0.

Table S3. Negative-database summary (ResFinder + NCBI AMR reference set)—environmental MBL-negative trio.

Table S4. ICEfinder analysis Job IDs and results for all submissions in this study.

Table S5. Per-allele acquired antimicrobial resistance gene consensus across the nine-genome *P. guariconensis*-clade panel.

Table S6. Pairwise average amino-acid identity (AAI) matrix across the nine-genome clade panel (3,197 Panaroo single-copy core orthologues).

File S1. Pre-WGS culture-purity check: taxonomic profiling of preliminary shotgun dataset (SRA SRR38121107).

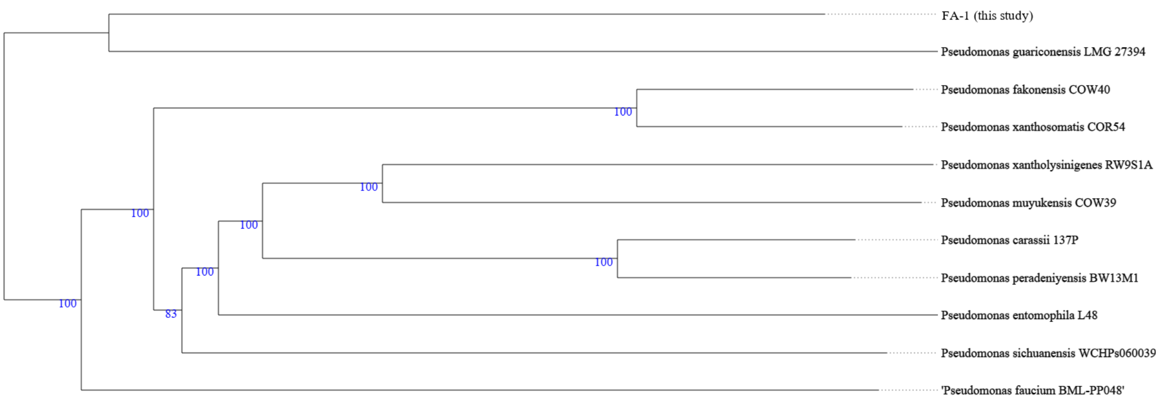

Figure S1. Genome BLAST Distance Phylogeny (GBDP) tree generated by the Type (Strain) Genome Server (TYGS), placing FA-1 (this study) among the closest *Pseudomonas* type-strain genomes identified by TYGS. The tree was inferred with FastME 2.1.6.1 from genome-based GBDP distances (formula d5), with branch lengths proportional to GBDP distance; node values are GBDP pseudo-bootstrap support (shown where >60%) from 100 replicates. FA-1 is placed nearest to the *P. guariconensis* type strain LMG 27394^{T} in this TYGS closest-type-strain context — consistent with its ANI (87.5–87.9%) and dDDH (32.9%; Section 3.1) — although pseudo-bootstrap support for the immediate FA-1–LMG 27394^{T} grouping is below 60%. TYGS assigned FA-1 to a separate species cluster, supporting candidate novel genomospecies status. This broader closest-type-strain framework complements the focused nine-genome core-genome maximum-likelihood phylogeny (Figure 2), which is restricted to the *P. guariconensis* clade for the resistance and mobile-element comparison.

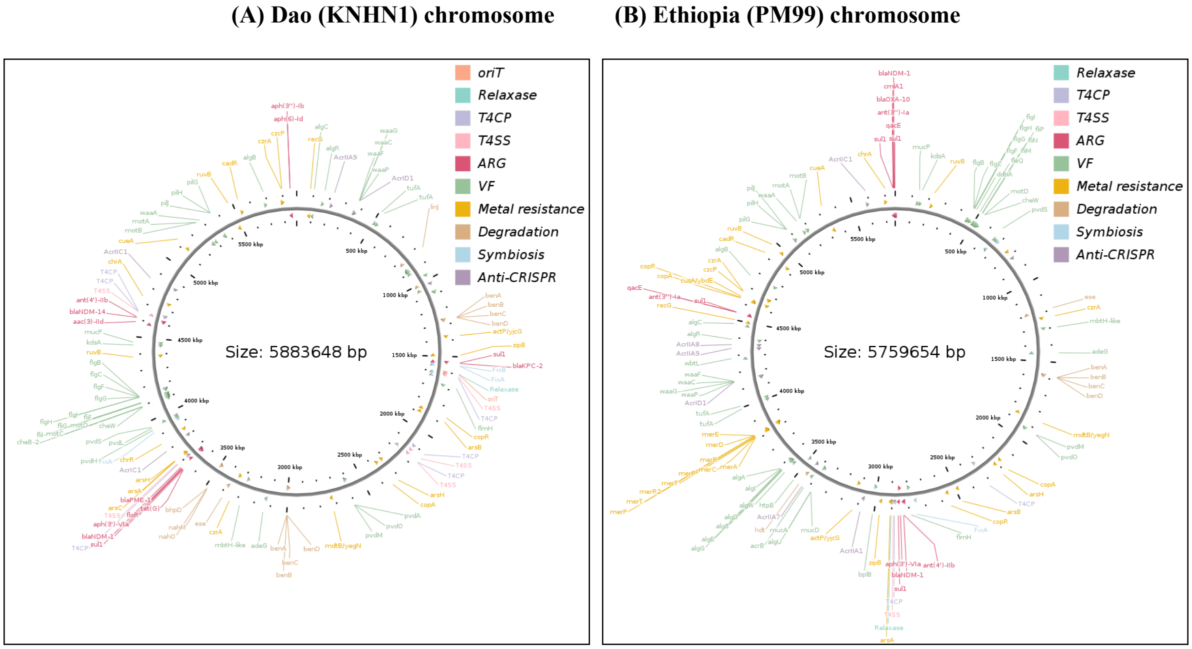

Figure S2. Conjugation-module maps predicted by oriTfinder2 (oriTDB) for (A) the Dao (KNHN1) chromosome (AP035765.1; 5,883,648 bp) and (B) the Ethiopia (PM99) chromosome (NZ_CP194068.1; 5,759,654 bp). Coloured markers denote the origin of transfer (oriT), relaxase, type IV coupling protein (T4CP), the type IV secretion system (T4SS) gene cluster, and accessory cargo modules including antimicrobial-resistance genes (ARG). The Dao chromosome carries a complete conjugative region (oriT, relaxase, T4CP and T4SS co-localised at ~1.60–1.63 Mb), whereas the Ethiopia chromosome carries the conjugation machinery (relaxase, T4CP and a T4SS gene cluster at ~2.88–2.90 Mb) without a detectable oriT. The marker tracks are shown for context and are not used for antimicrobial-resistance gene calling, which follows the ABRicate consensus described in Section 2.6.

Table S1. Reference genomes used in FastANI and phylogenomic analyses.

Organism	Strain	Role	Assembly Accession	Genome Size (Mb)	GC (%)	Isolation Source	Assembly level
Pseudomonas sp. FA-1	FA-1	This study (anchor, environmental)	JBWXTH010000000 (WGS; master JBWXTH000000000)	5.10	63.75	*Hyalomma dromedarii* internal tissues, Hail Province, Saudi Arabia	Draft WGS (38 contigs; N50 363 kb)
P. guariconensis	LMG 27394^T	Type strain (environmental)	GCF_900102675.1	5.08	62.60	*Zea mays* rhizosphere soil, Venezuela	Scaffold (N50 313 kb)
P. guariconensis	ABJ_B2_1	Environmental	GCF_044903045.1	4.98	62.68	Soil, Gwagwalada, Nigeria	Contig (N50 262 kb)
P. guariconensis	HY1196	Clinical	GCF_034724015.1	5.92	62.12	Urine, South Korea	Scaffold (N50 322 kb; scaffold N50 376 kb)
P. guariconensis	MGL_R1_GC	Clinical	GCF_036528595.1	5.34	62.41	Pleural fluid, Chhattisgarh, India	Scaffold (N50 322 kb; scaffold N50 441 kb)
P. guariconensis	GN-165	Clinical	GCF_036879405.1	5.12	62.54	Blood culture, Nuevo León, Mexico	Contig (N50 331 kb)
P. guariconensis	PM99	Clinical	GCF_051550215.1	5.83	62.09	Urine, Addis Ababa, Ethiopia	Complete genome (closed)
P. guariconensis	SAR321	Clinical	GCF_052077635.1	5.23	62.59	Wound, Nashville, TN, USA	Contig (1 contig, 5.23 Mb)
P. guariconensis	KNHN1 (Dao 2026)	Environmental, MBL-positive	AP035765.1 (chrom) + AP035766.1 (plasmid)	6.29	62.01	Hospital-downstream urban wastewater, Hanoi, Vietnam	Complete (chromosome + plasmid, both circular)

Table S2. Raw CARD database hits from ABRicate v1.4.0 (first-pass screen; all hits shown for transparency, including those below the consensus thresholds applied in Section 2.6 of the main text).

Contig	Gene	% Coverage	% Identity	Database	Reference accession
contig_11	Streptomyces_cinnamoneus_EF-Tu_mutants_conferring_resistance_to_elfamycin	16.50	76.88	card	X98831.1:362-1556
contig_12	MexE	91.73	78.07	card	NC_002516:2808743-2809988
contig_12	MexF	97.93	87.29	card	NC_002516:2810009-2813198
contig_12	OprN	94.01	77.57	card	NC_002516:2813194-2814613
contig_12	OprJ	93.55	78.10	card	U57969:4623-6063
contig_12	MexD	98.85	81.06	card	U57969:1486-4618
contig_12	MexC	91.42	77.13	card	U57969:295-1459
contig_135	Streptomyces_cinnamoneus_EF-Tu_mutants_conferring_resistance_to_elfamycin	12.90	77.56	card	X98831.1:362-1556
contig_14	MuxB	97.32	77.89	card	NC_002516.2:2850883-2854015
contig_1	OpmH	96.62	80.08	card	NC_002516.2:5584101-5585550
contig_2	mexK	98.83	82.08	card	AE004091.2:4116188-4119266
contig_4	MexA	83.61	75.67	card	NC_002516:472024-473176
contig_4	MexB	97.49	81.67	card	L11616:1570-4711
contig_4	OprM	95.07	77.46	card	NC_002516:476333-477791
contig_4	mexW	98.30	82.10	card	NC_002516.2:4904647-4907704
contig_5	*Pseudomonas*_aeruginosa_CpxR	99.71	81.18	card	LT673656.1:1884345-1885023
contig_5	*Pseudomonas*_aeruginosa_soxR	78.13	75.54	card	NC_002516.2:2503425-2503896
contig_8	TriC	99.02	78.90	card	NC_002516.2:179522-182570

Table S3. Negative-database summary for the environmental MBL-negative trio (FA-1, LMG 27394^{T}, ABJ_B2_1). Both ResFinder and the NCBI AMR Reference Gene Database (queried via ABRicate against the AMRFinderPlus reference set) returned zero acquired-resistance hits at the >=80% identity / >=80% coverage threshold; intrinsic *Pseudomonas* Mex-family efflux pumps were excluded per Methods, Section 2.6.

**Database**	**Version**	**Thresholds**	**Hits**	**Result**
ResFinder	4.0	80% identity, 80% coverage	0	No acquired-resistance genes detected
NCBI AMRFinderPlus	ABRicate default	80% identity, 80% coverage	0	No acquired-resistance determinants detected

Table S4. ICEfinder (ICEberg v2.0 database; https://tool2-mml.sjtu.edu.cn/ICEberg3/) Job IDs and results for all submissions performed in this study.

Submission ID	Input description	Length (bp)	Format	Job ID	Verdict
CTRL-1	*Klebsiella pneumoniae* HS11286 (CP003200.1; ICEfinder built-in reference)	5,333,942	(server)	VCFVX3Y5uo	2 T4SS-type ICEs (62.2 kb + 59.1 kb)
CTRL-2	ICEclc reference / AJ617740 (*Pseudomonas putida* B13)	105,032	FASTA	ScJjdE558k	1 T4SS-associated module (24.1 kb, 50,246-74,302)
ETH-CASS-A	Ethiopia NDM-1 #1 cassette (FASTA, dual-format validation)	25,000	FASTA	PuKe9En3c8	No ICE
ETH-CASS-B	Ethiopia Section 3.5 cassette (NZ_CP194068.1:5,740,000-5,759,654)	19,654	FASTA	lcq5duHdss	No ICE, Section 3.5 cassette
ETH-CHROM	Ethiopia full chromosome (NZ_CP194068.1)	5,759,654	FASTA	xQ52e0OWUk	1 T4SS-type ICE at 2,880,460-2,904,589 (24,130 bp)
DAO-CASS-1	Dao 2026 NDM-1 cassette (AP035765.1:3,660,000-3,685,000)	25,000	FASTA (revalidation 2026-05-20)	wEWMBz5OvV	No ICE in 25 kb cassette window
DAO-CASS-2	Dao 2026 AFM-5 cassette (AP035765.1:4,598,000-4,623,000)	25,000	GBK (revalidation 2026-05-20)	LwBVQWwjfy	No ICE
DAO-CASS-2	Dao 2026 AFM-5 cassette FASTA	25,000	FASTA	ZFB2xDIAls	No ICE
DAO-CASS-3	Dao 2026 KPC-2 cassette (AP035765.1:1,525,000-1,550,000)	25,000	GBK	kiBiZ5DKqe	No ICE
DAO-CASS-3	Dao 2026 KPC-2 cassette FASTA	25,000	FASTA (revalidation 2026-05-20)	bUmUFnRiLy	No ICE
DAO-CHROM	Dao 2026 full chromosome (AP035765.1; integrated into nine-genome panel)	5,883,648	FASTA	rrVozGYfMQ	4 T4SS-type ICEs detected (44 + 101 + 24 + 31 kb)

Notes. ETH-CASS-A and ETH-CASS-B are two distinct chromosomal NDM-1 contexts on the same Ethiopia replicon separated by ~2.9 Mb (see Section 3.5). The cassette referenced in Section 3.5 of the main manuscript corresponds to ETH-CASS-B (lcq5duHdss). The full-chromosome ICE prediction (ETH-CHROM, xQ52e0OWUk) is adjacent to the ETH-CASS-A region. Dao 2026 (KNHN1, AP035765.1) is included as the environmental MBL-positive wastewater comparator in the nine-genome panel (DAO-CHROM full-chromosome ICEfinder run). The GBK format error encountered on the ETH-CASS-B initial submission (daNA5Ua4qq, not shown) was resolved by FASTA resubmission (lcq5duHdss); GBK and FASTA submissions of the same locus returned identical biological verdicts where both succeeded. Job IDs in the table correspond one-to-one with archived PDFs in the verified primary-data cache; revalidation submissions (2026-05-20) replaced original Dao cassette submissions and are the canonical entries.

Table S5. Per-allele acquired antimicrobial resistance gene consensus across the nine-genome *Pseudomonas guariconensis*-clade panel (ABRicate v1.4.0 against CARD, NCBI AMRFinderPlus, ResFinder, and ARG-ANNOT databases).

Genome	Replicon	Position (start–end)	Gene (canonical)	Allele	Identity (%)	Class	Notes
KNHN1 (Dao)	AP035765.1 (chrom)	1,537,989 – 1,538,870	blaKPC-2	KPC-2	100	Class A serine carbapenemase	Hotspot α; carbapenemase
KNHN1 (Dao)	AP035765.1 (chrom)	~1,533,000 (Hotspot α)	sul1	sul1	100	Sulfonamide	Hotspot α; class 1 integron marker
KNHN1 (Dao)	AP035765.1 (chrom)	Hotspot β (3.65–3.68 Mb)	tet(G)	tet(G)	100	Tetracycline efflux	Hotspot β
KNHN1 (Dao)	AP035765.1 (chrom)	Hotspot β (3.65–3.68 Mb)	floR	floR2	100	Phenicol efflux	Hotspot β
KNHN1 (Dao)	AP035765.1 (chrom)	Hotspot β (3.65–3.68 Mb)	aph(3′)-VIa	aph(3′)-VIa	98.08	Aminoglycoside phosphotransferase	Hotspot β
KNHN1 (Dao)	AP035765.1 (chrom)	3,668,228 – 3,669,157	blaPME-1	PME-1	100	Class A ESBL (NOT carbapenemase)	Hotspot β; cephalosporin-only
KNHN1 (Dao)	AP035765.1 (chrom)	3,671,399 – 3,672,211	blaNDM-1	NDM-1	100	Class B1 MBL	Hotspot β; carbapenemase
KNHN1 (Dao)	AP035765.1 (chrom)	~3,678,000 (Hotspot β)	sul1	sul1	100	Sulfonamide	Hotspot β; class 1 integron marker
KNHN1 (Dao)	AP035765.1 (chrom)	Hotspot γ (4.59–4.61 Mb)	tmexC	tmexC2	98.37	RND efflux periplasmic subunit	Hotspot γ; Dao allele variant
KNHN1 (Dao)	AP035765.1 (chrom)	Hotspot γ (4.59–4.61 Mb)	tmexD	tmexD2	100	RND efflux transporter	Hotspot γ; Dao allele variant
KNHN1 (Dao)	AP035765.1 (chrom)	Hotspot γ (4.59–4.61 Mb)	toprJ	toprJ2	99.93	RND efflux outer-membrane factor	Hotspot γ; Dao allele variant
KNHN1 (Dao)	AP035765.1 (chrom)	Hotspot γ (4.59–4.61 Mb)	ble-MBL	BRP(MBL)	89.07	Bleomycin resistance protein	Hotspot γ
KNHN1 (Dao)	AP035765.1 (chrom)	4,610,546 – 4,611,349	blaAFM-5	AFM-5	100	Class B1 MBL (Alcaligenes faecalis MBL family)	Hotspot γ; carbapenemase (corrects an earlier Prokka 'duplicate NDM' annotation)
KNHN1 (Dao)	AP035765.1 (chrom)	Hotspot γ (4.59–4.61 Mb)	ant(4′)-IIb	ant(4′)-IIb	99.60	Aminoglycoside nucleotidyltransferase	Hotspot γ
KNHN1 (Dao)	AP035765.1 (chrom)	ABRicate ≥80%/80%	aph(3″)-Ib (strA)	aph(3″)-Ib	100	Aminoglycoside phosphotransferase	Per ABRicate consensus 3/4 DBs (argannot+ncbi+resfinder); 100% identity, 100% coverage; integron-cassette-associated
KNHN1 (Dao)	AP035765.1 (chrom)	ABRicate ≥80%/80%	aph(6)-Id (strB)	aph(6)-Id	99.9	Aminoglycoside phosphotransferase	Per ABRicate consensus 3/4 DBs; 99.9% identity, 100% coverage; integron-cassette-associated
KNHN1 (Dao)	AP035765.1 (chrom)	ABRicate ≥80%/80%	aac(3)-IId	aac(3)-IId	99.9	Aminoglycoside N-acetyltransferase	Per ABRicate consensus 2/4 DBs (ncbi+resfinder); 99.9% identity, 100%

							coverage; integron-cassette-associated
KNHN1 (Dao)	AP035765.1 (chrom)	ABRicate ≥80%/80 %	aadA1 (ant(3″)-Ia)	aadA1	99.9	Aminoglycoside adenylyltransferase	Per ABRicate consensus 2/4 DBs (ncbi+resfinder); 99.9% identity, 99.3% coverage; class 1 integron marker
PM99 (Ethiopia)	NZ_CP194068.1 (chrom)	2,828,332 – 2,829,086	ant(4′)-IIb	ant(4′)-IIb	89.4	Aminoglycoside nucleotidyltransferase	Cassette A (lonely NDM-1)
PM99 (Ethiopia)	NZ_CP194068.1 (chrom)	2,831,950 – 2,832,762	blaNDM-1	NDM-1 (upstream copy)	100	Class B1 MBL	Cassette A (lonely NDM-1); 50 kb downstream from ICE Job xQ52e0OWUk
PM99 (Ethiopia)	NZ_CP194068.1 (chrom)	2,849,709 – 2,850,488	aph(3′)-VIa	aph(3′)-VIa	98.1	Aminoglycoside phosphotransferase	Cassette A
PM99 (Ethiopia)	NZ_CP194068.1 (chrom)	2,861,852 – 2,862,691	sul1	sul1	100	Sulfonamide	Cassette A
PM99 (Ethiopia)	NZ_CP194068.1 (chrom)	5,746,986 – 5,747,825	sul1	sul1 (copy 1)	100	Sulfonamide	Cassette B super-cassette
PM99 (Ethiopia)	NZ_CP194068.1 (chrom)	5,748,330 – 5,749,121	aadA1	aadA1	100	Aminoglycoside adenylyltransferase	Cassette B
PM99 (Ethiopia)	NZ_CP194068.1 (chrom)	5,749,230 – 5,750,030	blaOXA-10	OXA-10	100	Class D β-lactamase (narrow-spectrum; NOT carbapenemase)	Cassette B
PM99 (Ethiopia)	NZ_CP194068.1 (chrom)	5,750,295 – 5,751,554	cmlA	cmlA1	100	Chloramphenicol exporter	Cassette B
PM99 (Ethiopia)	NZ_CP194068.1 (chrom)	5,753,659 – 5,754,024	ble-MBL	BRP(MBL)	100	Bleomycin resistance protein	Cassette B
PM99 (Ethiopia)	NZ_CP194068.1 (chrom)	5,754,028 – 5,754,840	blaNDM-1	NDM-1 (cassette copy)	100	Class B1 MBL	Cassette B; ~4 kb from OXA-10
PM99 (Ethiopia)	NZ_CP194068.1 (chrom)	5,757,127 – 5,757,966	sul1	sul1 (copy 2)	100	Sulfonamide	Cassette B
PM99 (Ethiopia)	NZ_CP194068.1 (chrom)	5,758,476 – 5,759,030	aac(6′)-Ib	aac(6′)-Ib4	100	Aminoglycoside N-acetyltransferase	Cassette B
PM99 (Ethiopia)	NZ_CP194068.1 (chrom)	5,759,169 – 5,759,621	arr-3	arr-3	100	Rifampicin ADP-ribosyltransferase	Cassette B
PM99 (Ethiopia)	NZ_CP194069.1 (plasmid)	168–1,007	sul1	sul1	100	Sulfonamide	Plasmid
PM99 (Ethiopia)	NZ_CP194069.1 (plasmid)	3,216–3,872	qnrVC6	qnrVC6	100	Fluoroquinolone resistance	Plasmid
PM99 (Ethiopia)	NZ_CP194069.1 (plasmid)	11,478–12,011	dfrA42	dfrA42	100	Trimethoprim dihydrofolate reductase	Plasmid
PM99 (Ethiopia)	NZ_CP194069.1 (plasmid)	68,142–68,942	blaVIM-4	VIM-4	100	Class B1 MBL	Plasmid; only plasmid-borne carbapenemase in nine-genome panel
PM99 (Ethiopia)	NZ_CP194069.1 (plasmid)	69,036–69,494	aac(6′)-Il	aac(6′)-Il	100	Aminoglycoside N-acetyltransferase	Plasmid
HY1196 (Korea)	NZ_JAXLOJ010000042.1 (small contig; likely plasmid)	624–1,424	blaVIM-2	VIM-2	100	Class B1 MBL	Small contig, likely plasmid-associated
HY1196 (Korea)	(multi-contig)	ABRicate ≥80%/80	strA / aph(3″)-Ib	aph(3″)-Ib	≥80% (consensu	Aminoglycoside phosphotransferas	Per ABRicate consensus

		%			s)	e	
HY1196 (Korea)	(multi-contig)	ABRicate ≥80%/80 %	strB / aph(6)-Id	aph(6)-Id	≥80% (consensus)	Aminoglycoside phosphotransferase	Per ABRicate consensus
HY1196 (Korea)	(multi-contig)	ABRicate ≥80%/80 %	aac(3)-Ib/If	aac(3)-Ib/If	≥80% (consensus)	Aminoglycoside N-acetyltransferase	Per ABRicate consensus
HY1196 (Korea)	(multi-contig)	ABRicate ≥80%/80 %	aac(6′)-Ib	aac(6′)-Ib variants	≥80% (consensus)	Aminoglycoside N-acetyltransferase	Per ABRicate consensus; shared with Ethiopia
MGL_R1_GC (India)	Chromosomal	ABRicate ≥80%/80 %	tmexC	tmexC1	≥80% (consensus)	RND efflux periplasmic subunit	tmexCD1-toprJ1; distinct from Dao tmexC2D2-toprJ2
MGL_R1_GC (India)	Chromosomal	ABRicate ≥80%/80 %	tmexD	tmexD1	≥80% (consensus)	RND efflux transporter	tmexCD1-toprJ1
MGL_R1_GC (India)	Chromosomal	ABRicate ≥80%/80 %	toprJ	toprJ1	≥80% (consensus)	RND efflux outer-membrane factor	tmexCD1-toprJ1

Notes.

1. Dao chromosomal resistance genes are organised into three IS-bounded resistance hotspots (α at 1.54 Mb; β at 3.65–3.68 Mb; γ at 4.59–4.61 Mb). The blaAFM-5 call at 100% identity supersedes an earlier Prokka first-pass annotation that reported the locus as a divergent NDM-like at ~91% identity to NDM-14; ABRicate-NCBI at 100% identity to NG_242697.1 (AFM-5 reference) is definitive.

2. Ethiopia chromosomal blaNDM-1 is present at two distinct loci: the upstream NDM-1 copy in Cassette A (the lonely NDM-1 cassette around 2.83 Mb, ~50 kb downstream of the ICEfinder-predicted T4SS-encoding ICE at 2,880,460–2,904,589) and the cassette NDM-1 copy in Cassette B (the multi-resistance super-cassette around 5.75 Mb, within ~4 kb of blaOXA-10).

3. blaOXA-10 is a narrow-spectrum class D β-lactamase that does not hydrolyse carbapenems at clinically relevant levels; it is included in the matrix because it is an acquired β-lactamase but is NOT counted among the carbapenemase loci reported in the main text. blaPME-1 is a class A extended-spectrum β-lactamase (cephalosporin-only); also included but not a carbapenemase.

4. The Dao tmexC2 + tmexD2 + toprJ2 alleles are distinct from the India tmexC1 + tmexD1 + toprJ1 alleles at the allele level. Main-text Table 2 records both under the same canonical gene rows (tmexC / tmexD / toprJ) for presence/absence purposes; this supplementary table records the allele-level difference.

5. The Korea HY1196 VIM-2 contig is small (NZ_JAXLOJ010000042.1; 1.4 kb in the reported window) and consistent with a plasmid origin. The remaining Korea aminoglycoside resistance genes (strA / strB / aac(3)-Ib/If / aac(6′)-Ib) are recorded at the ABRicate-validated presence/absence level but were not extracted at the per-allele position resolution for this study.

6. AMR-negative genomes (FA-1, LMG 27394ᵀ type strain, ABJ_B2_1 Nigerian soil, GN-165 Mexico, SAR321 USA-Nashville) returned zero acquired-resistance hits at the consensus threshold. As shown in Table S2, raw CARD searches return hits at permissive screening parameters (%identity 75.5-87.3%, %coverage 12.9-99.7%; Table S2) against the intrinsic *Pseudomonas* Mex-family efflux pumps (MexAB-OprM, MexCD-OprJ, MexEF-OprN orthologues), OpmH, MuxB, TriC, and chromosomal regulators (CpxR, SoxR-like); per Methods §2.6 these intrinsic loci are present in all nine genomes and were excluded from the acquired-resistance matrix to isolate horizontally acquired resistance. PlasmidFinder was screened separately for plasmid replicons.

7. Allele consolidation and database-support thresholds: aphA-6 is reported under aph(3′)-VIa, as this table (Table S5) records only aph(3′)-VIa for the Ethiopia chromosomal locus (2,849,709–2,850,488) and no separate aphA-6 physical locus is present. qacEΔ1 is supported by a single AMR-gene database only (ARG-ANNOT at ≥80/80) and is therefore excluded under the ≥2-of-4 database consensus rule applied throughout this matrix. Per-isolate consolidated unique-gene-label counts: Dao = 17, Ethiopia = 14, Korea = 5, India = 3, FA-1 = 0, LMG 27394ᵀ = 0, ABJ_B2_1 (Nigerian soil) = 0, GN-165 (Mexico) = 0, SAR321 (USA-Nashville) = 0.

8. Cassette nomenclature: the Ethiopia (PM99) regions labelled Cassette A and Cassette B in this table correspond, respectively, to the ICEfinder submissions ETH-CASS-A (bla*NDM-1* upstream copy, ≈2.83 Mb) and ETH-CASS-B (the Section 3.5 super-cassette, ≈5.75 Mb) in Table S4.

Table S6. Pairwise average amino-acid identity (AAI, %) across the nine-genome *Pseudomonas guariconensis*-clade panel, computed as the mean amino-acid identity over 3,197 Panaroo single-copy core orthologues (global Needleman–Wunsch alignment, BLOSUM62; identity over aligned non-gap columns). Diagonal entries are 100 by definition. The seven *sensu stricto* genomes (Type strain, Dao, Ethiopia, India, Mexico, Nigeria, USA-Nashville) share 99.34–99.83% pairwise AAI; HY1196 averages 97.15% and FA-1 93.57% to that cluster.

	**Dao**	**FA-1**	**Type strain**	**Ethiopia**	**India**	**HY1196**	**Mexico**	**Nigeria**	**USA-Nashville**
**Dao**	100	93.57	99.77	99.75	99.79	97.14	99.36	99.35	99.79
**FA-1**	93.57	100	93.57	93.56	93.58	93.53	93.57	93.57	93.58
**Type strain**	99.77	93.57	100	99.77	99.77	97.14	99.35	99.34	99.78
**Ethiopia**	99.75	93.56	99.77	100	99.76	97.13	99.37	99.38	99.76
**India**	99.79	93.58	99.77	99.76	100	97.15	99.37	99.35	99.82
**HY1196**	97.14	93.53	97.14	97.13	97.15	100	97.16	97.16	97.15
**Mexico**	99.36	93.57	99.35	99.37	99.37	97.16	100	99.83	99.38
**Nigeria**	99.35	93.57	99.34	99.38	99.35	97.16	99.83	100	99.36
**USA-Nashville**	99.79	93.58	99.78	99.76	99.82	97.15	99.38	99.36	100

File S1. Pre-WGS culture-purity check: taxonomic profiling of preliminary shotgun dataset (SRA SRR38121107).

The preliminary shotgun dataset (SRA SRR38121107) was generated in the upstream workflow from the same cultured-isolate DNA as the full WGS run (SRR38121108) and served as an orthogonal pre-WGS culture-purity check; it was profiled with two complementary tools for lineage-level taxonomic context.

Kraken2 v2.1 (PlusPFP database): Kraken2 classified 96.0% of classified shotgun reads to the genus *Pseudomonas*. The dominant species-level label was a *Pseudomonas* sp. of the *P. guariconensis* lineage ("*Pseudomonas* sp. p1(2021b)"). Full Kraken2 report statistics: 67.09% of total reads classified (26,721,328 / 39,831,549); 32.91% unclassified. The *P. putida* group accounted for 33.30% of total reads (13,262,076 / 39,831,549), corresponding to 49.6% of classified reads (13,262,076 / 26,721,328), with the largest single fraction mapping to *P. putida sensu lato*.

MetaPhlAn4 (mpa_vJun23_CHOCOPhlAnSGB_202403 database): MetaPhlAn4 placed the dominant *Pseudomonas* signal in species-genome-bin SGB12273, associated with *P. guariconensis*.

These assignments are consistent with the cultured FA-1 assembly's taxonomic placement within the *P. guariconensis* lineage.

Raw shotgun reads are deposited in NCBI SRA under BioProject PRJNA1449514 and BioSample SAMN57124393.
